# Distinguishing host responses, extensive viral dissemination and long-term viral RNA persistence in domestic sheep experimentally infected with Crimean-Congo haemorrhagic fever virus Kosovo Hoti

**DOI:** 10.1080/22221751.2024.2302103

**Published:** 2024-01-08

**Authors:** Hongzhao Li, Mathieu Pinette, Greg Smith, Melissa Goolia, Katherine Handel, Michelle Nebroski, Oliver Lung, Bradley S. Pickering

**Affiliations:** a National Centre for Foreign Animal Disease, Canadian Food Inspection Agency, Winnipeg, Canada; b Department of Medical Microbiology and Infectious Diseases, College of Medicine, Faculty of Health Sciences, University of Manitoba, Winnipeg, Canada

**Keywords:** Crimean-Congo haemorrhagic fever virus, Kosovo Hoti, sheep, virus shedding, RNA persistence

## Abstract

Crimean-Congo haemorrhagic fever orthonairovirus (CCHFV) is a tick-borne, risk group 4 pathogen that often causes a severe haemorrhagic disease in humans (CCHF) with high case fatality rates. The virus is believed to be maintained in a tick-vertebrate-tick ecological cycle involving numerous wild and domestic animal species; however the biology of CCHFV infection in these animals remains poorly understood. Here, we experimentally infect domestic sheep with CCHFV Kosovo Hoti, a clinical isolate representing high pathogenicity to humans and increasingly utilized in current research. In the absence of prominent clinical signs, the infection leads to an acute viremia and coinciding viral shedding, fever and markers for potential impairment in liver and kidney functions. A number of host responses distinguish the subclinical infection in sheep versus fatal infection in humans. These include an early reduction of neutrophil recruitment and its chemoattractant, IL-8, in the blood stream of infected sheep, whereas neutrophil infiltration and elevated IL-8 are features of fatal CCHFV infections reported in immunodeficient mice and humans. Several inflammatory cytokines that correlate with poor disease outcomes in humans and have potential to cause vascular dysfunction, a primary hallmark of severe CCHF, are down-regulated or restricted from increasing in sheep. Of particular interest, the detection of CCHFV RNA (including full-length genome) in a variety of sheep tissues long after the acute phase of infection indicates a widespread viral dissemination in the host and suggests a potentially long-term persisting impact of CCHFV infection. These findings reveal previously unrecognized aspects of CCHFV biology in animals.

## Introduction

CCHFV is a risk group 4 pathogen characterized by high case fatality rates (CFR) in humans ranging from 5% to 30% but up to 80% in some outbreaks [1–4] and an absence of internationally licensed vaccines or therapeutics. The virus has been listed by the World Health Organization as a top priority pathogen of epidemic and pandemic potential in urgent need for research and development [5,6]. Belonging to the genus *Orthonairovirus*, family *Nairoviridae*, order *Bunyavirales* [7], CCHFV is an enveloped virus with an RNA genome consisting of the small (S), medium (M), and large (L) segments, which encode the nucleoprotein, glycoprotein precursor and RNA-dependent RNA polymerase, respectively [1]. Ixodid (hard-body) ticks from the genus *Hyalomma* are the principle reservoir and vector for the virus and human infections most often occur through tick exposure [1]. CCHFV has a widespread geographic distribution with a recent trend of expansion and re-emergence (detailed in Introduction S1).

In humans CCHFV infection can result in a range of disease outcomes, including a severe, often fatal, haemorrhagic fever disease (CCHF). Several correlates or predictors of poor outcomes were identified (Introduction S1). In wild and domestic vertebrate species CCHFV infection does not appear to produce prominent disease. A few exceptions exist in laboratory animals (Introduction S1). Experimental infection studies, together with serosurveys, have largely established a tick-vertebrate-tick ecological cycle for CCHFV maintenance and transmission. In addition to their role in feeding, carrying and transporting ticks, a variety of animal species serve as viral amplification hosts. However, numerous gaps remain in the research of CCHFV infection in animals. Past experimental studies were reported during the period of 1945–1978, many in the Russian-language literature, with only six published more recently (1980s–1990s) [1,8,9]. It has been noted that findings from these studies remain to be validated using modern laboratory methods [1]. Conclusions from these studies may also have been confounded by variations in inoculum source, which was based on viral strains of unclear pathogenicity or poor characterization [9]. Data from experimental infections of animals have so far been limited to the assessment of viremia and, in some studies, antibody responses, without in-depth clinical and biological findings [9]. Host responses associated with disease control, viral shedding and viral dissemination and persistence in tissues of animals are notable gaps in CCHFV knowledge [9]. Novel insights gained by addressing these questions are anticipated to identify candidate targets for medical countermeasure development and provide critical guidance for public health education and interventions in One Health management.

In the current study, we conducted experimental infections of domestic sheep using the Kosovo Hoti strain of CCHFV. Originally isolated from a fatal human case from an endemic region with high CFR [10], the strain represents high human pathogenicity and is increasingly used in current research toward CCHFV medical countermeasures as well as in pathogenicity studies [11–15]. Our detailed analysis of virological, clinical, and immunological parameters associated with CCHFV infection in sheep identified differential host responses contrasting those known for fatal infections in humans and immunodeficient mouse models. Further, this work provided evidence of viral shedding concerning different routes, widespread viral dissemination in the host and long-term viral RNA persistence in tissues.

## Materials and methods

In addition to the brief summary of the core materials and methods below, detailed information on all materials and methods can be found in Materials and Methods S1.

### Experimental infection of sheep with CCHFV

Four domestic sheep (*Ovis aries*) of the Rideau-Arcott breed, male and approximately two months old, were sourced from a farm in Manitoba, Canada and used for infection with CCHFV. These animals were named Sheep 21-01, 21-02, 21-03, and 21-04. 10 uninfected sheep remaining in the same farm flock as the four sheep for infection originated from, with the same/similar biological characteristics (breed, sex, and age), were involved in blood and swab sampling to provide additional control samples, but were not challenged with the virus or sacrificed. These uninfected sheep were only subjected to sampling once and no complications would be expected to occur following this sampling event. The four animals undergoing experimental infection were housed together (with possibility of viral transmission among animals). Each animal was injected with CCHFV Kosovo Hoti via both the subcutaneous (under the skin just behind or in front of the shoulder blade) and intravenous (into the jugular vein) routes at 1.2 × 10^6^ TCID50/route. A daily visual check of apparent wellbeing was performed between −1 and 34 days post infection (DPI), the baseline and study endpoint days, respectively. Recording of clinical signs of illness and collection of blood and swabs (nasal, oral, and rectal) was conducted daily on −1, 1–10, 14, 21, 28, and 34 DPI, except that during 1–10 DPI, two animals were subject to blood and swab sampling every other day (Sheep 21-01 and 21-03 on odd number DPI, whereas 21-02 and 21-04 on even number DPI). The alternate day sampling was initiated to reduce the distress on the animals so as not to subject them to every day sampling for 10 consecutive days. On 34 DPI, after routine sampling all animals were euthanized and the following organs/tissues were collected: skeletal muscle – right quadriceps, inguinal lymph nodes, gastrohepatic lymph nodes, liver, spleen, mesenteric lymph nodes, ileum, adrenal gland, kidney, lung – right middle lobe, heart, tracheobronchial lymph nodes, deep cervical lymph nodes, cerebrum, cerebellum, hypothalamus, testicle, lung – left cranial lobe, additional lung tissue and cerebrospinal fluid.

To address the clinical and biological responses to CCHFV infection in the four challenged sheep, changes were analyzed between the baseline (before infection) and subsequent time point of interest (after infection), or in other words, between the “baseline control group” and “infected group.” The smallest sample size followed the 3Rs principles and was calculated using the UCSF Sample Size Calculators for designing clinical research [16]. This was based on a type 1 error, α (two-tailed) = 0.05, and a type 2 error, β = 0.2, the proportion of subjects that are in group 0 (unexposed), q0 = 0.5, the proportion of subjects that are in group 1 (exposed), q1 = 0.5, risk in group 0 (baseline risk), P0 = 0.001 and risk in group 1 (exposed), P1 = 0.9. The estimated results indicated no false positives or false negatives. To enhance statistical power, an enlarged negative control group (NC) of 14 sheep was formed by combining the four sheep for infection at the baseline state with the 10 additional uninfected sheep. Thus, an extended analysis was performed comparing samples from the enlarged NC group and samples from the infected sheep group.

## Results and discussion

### Susceptibility of sheep to CCHFV infection

**Figure 1.**
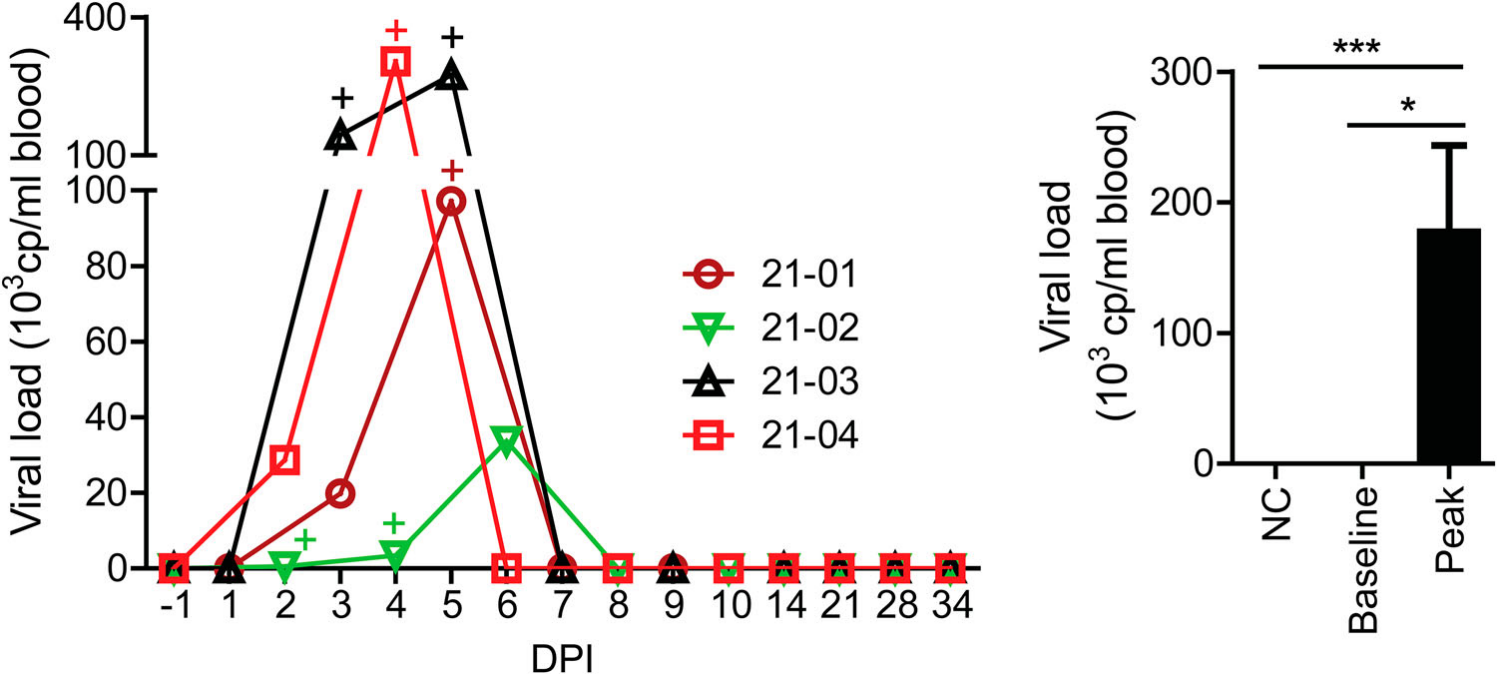
Viremia in CCHFV-infected sheep. Viremia, indicated as viral load in whole blood, was measured as viral RNA copy (cp) number/ml blood by RT-qPCR (limit of detection 500 cp/ml blood). Line graph shows the viral load time course for each experimentally infected animal. The “+” symbol marks the time points where infectious virus was isolated. Bar graph (mean ± SEM) compares viral loads at their peak in the infected animals against those at the baseline (−1 DPI) or those in 14 uninfected, negative control (NC) animals. The induction of viremia by CCHFV infection was found statistically significant: *p < 0.05 and ***p < 0.001.

Our study design included factors previously assessed for correlation with seroprevalence of CCHFV antibodies in both animals and humans. While sex showed no effect in sheep [17], increasing age was associated with higher antibody prevalence in sheep, other domestic animals and humans [17–27]. Age may reflect the amount of exposure of livestock or humans to infected ticks [23]. Alternatively, cases in younger aged animals may be linked to higher resistance to infection. In newborn mice, however, younger age appears to confer higher susceptibility to CCHF disease. Taking the potential effect of age into account, we focused this study on characterizing CCHFV infection in sheep of younger age. Four male lambs at approximately two months of age, identified as Sheep 21-01, 21-02, 21-03, and 21-04, respectively, were each challenged with CCHFV Kosovo Hoti through both the subcutaneous and intravenous routes (1.2 × 10^6^ TCID50 per route). Animals were monitored for clinical signs daily and sampled on alternate days or weekly. Viremia (or viral load), as measured by viral RNA in whole blood, was detected in all animals, at time points ranging from 2 DPI to 6 DPI, with peak levels observed around 5 DPI and followed by a quick decline to undetectable levels (Figure 1). We performed virus isolation on blood samples covering time points from −1 DPI (baseline) to 8 DPI. Infectious virus was isolated in all animals, from the time points within the viremic period (2 DPI–6 DPI) (Figure 1). For virus isolation, each blood sample was subjected to the following dilutions: undiluted or diluted at 1:10, 1:100, or 1:1000. 100 µl of these diluted samples was each applied to testing. Among these, viral isolation was successful at down to 1:100 dilution but not at 1:1000 dilution, indicating that the calculated sensitivity of the virus isolation is in the order of 1000 infectious units/ml. These results demonstrate that lambs are susceptible to CCHFV infection. It is noteworthy that the magnitude of viremia, or viral (RNA) load, did not always correlate with that of infectious virus, and CCHFV infectivity appeared to be highly efficient, even at very low viral RNA concentrations. These are discussed in Results and discussion S1.

### Clinical signs, haematological features and biochemical characteristics

While no major disease was apparent (Tables S1–S4), all animals consistently demonstrated a significant increase in body temperatures following infection (Tables S1–S4 and Figure S1). We measured rectal temperatures and defined those above 39.9°C as higher than normal body temperatures (fever) according to the literature [28,29]. Fever was the most dramatic during the viremic period, with peak temperatures (above 41°C in all animals) largely occurring on or near 5 DPI, coinciding with peak viremia (Figure 1). Interestingly, elevated temperatures were also observed after the viremic period (Figure S1). It is unclear whether these resulted from a persistent effect of CCHFV infection; however, a prolonged fever in Sheep 21-04 and a late fever spike in Sheep 21-01 appeared to be associated with high levels of persistent viral RNA in tissues (see below).

**Figure 2.**
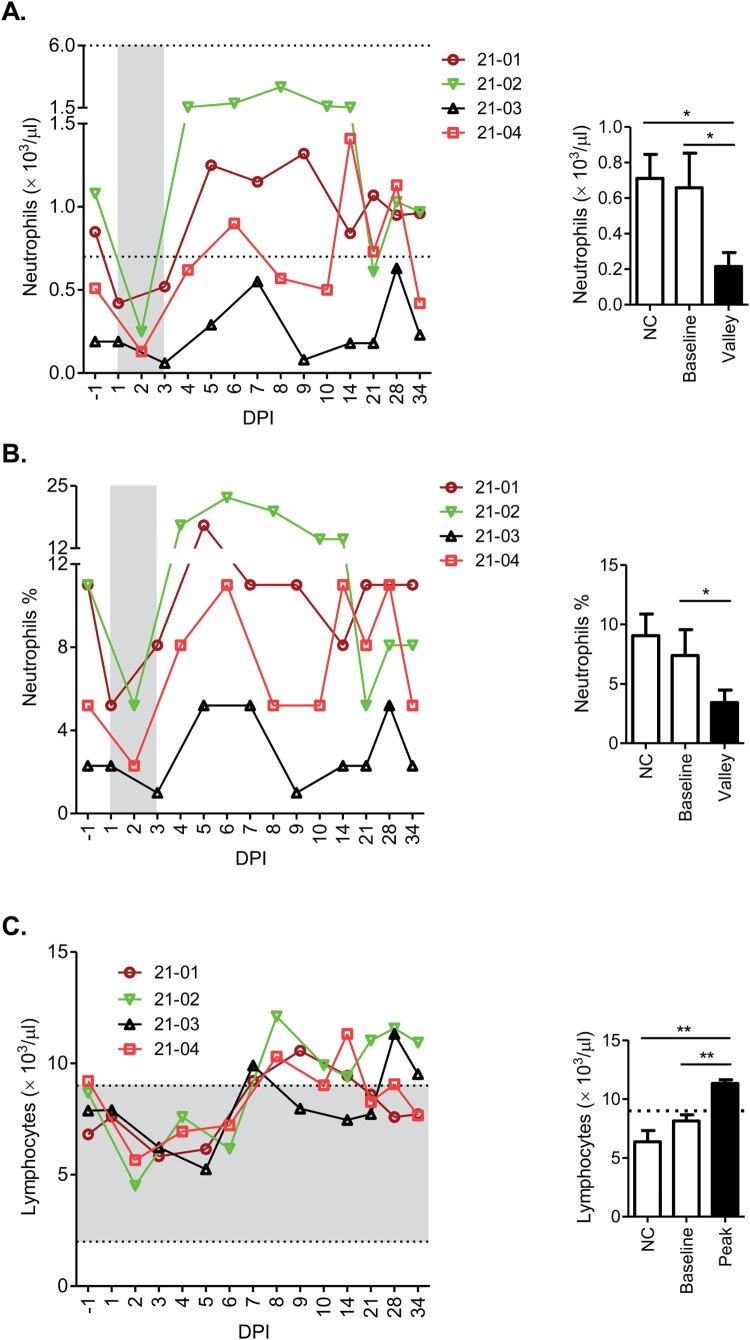
Haematological changes following CCHFV infection. Data on neutrophil count (A), neutrophil % within the leucocyte population (B) and lymphocyte count (C) are presented in graph formats similar to those in Figure 1. Grey columns in A and B highlight window of an early reduction of neutrophil count or percentage to a bottom level consistently found in all animals. For contrasting purpose, double dash lines in A and C indicate the upper and lower limits of normal parameter ranges commonly observed for sheep in the literature, and single dash line in C indicates the upper limit.

Haematological changes in total white blood cells, monocytes, red blood cells or platelets were not found (Tables S1–S4). Consistent with the normal platelet parameters, prothrombin time and activated partial thromboplastin time also showed no change (Tables S1–S4). However, all animals demonstrated a sharp reduction in neutrophil count and percentage (relative to total white blood cells) in the time window of 1 DPI to 3 DPI, followed by a rebound to levels higher than the baseline and return to near the baseline at later time points (Figure 2A,B). The role of neutrophils in the control or pathogenesis of CCHFV infection is largely unknown. According to previous findings in CCHF and other disease models, we hypothesize that neutrophils may exert a pathological effect on susceptible hosts toward fatal CCHF disease, and sheep may evade or counter such effect by restricting the recruitment of neutrophils into the blood stream (detailed discussion in Results and discussion S1). Moreover, a significant spike in lymphocyte count was detected following peak viremia, at time points ranging from 7 DPI to 9 DPI (Figure 2C), possibly reflecting the expansion of lymphocytes during advancing adaptive immune responses. The maintenance and expansion of lymphocytes in sheep represents a distinctive feature as lymphopenia is a hallmark of severe CCHF in humans, cynomolgus macaques and immunodeficient mice [12,30,31]. Additionally, analyzes of blood biochemical markers for liver and kidney disease identified changes that imply a transient and minor impairment in certain biochemical functions of the liver and kidney (Results and discussion S1; Figure S2).

### Immunological findings

**Figure 3.**
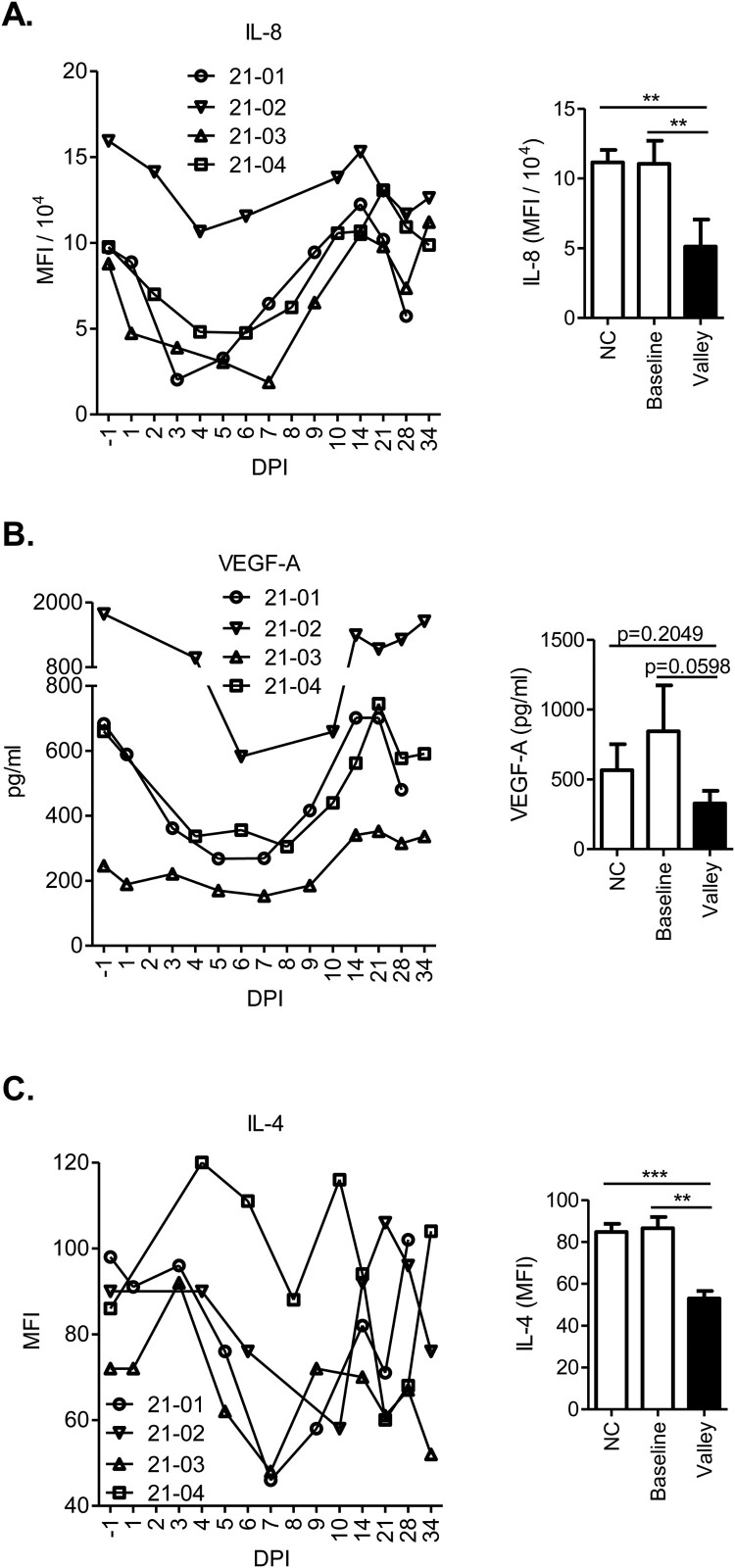
Cytokines that demonstrated decreases following CCHFV infection. Serum levels of cytokines were measured by Luminex assay. Line graphs show the cytokine time course for each experimentally infected animal. Bar graphs (mean ± SEM) compare cytokine concentrations at their lowest level (Valley) in the infected animals against those at the baseline (−1 DPI) or those in 14 uninfected, negative control (NC) animals. Decreases in these cytokines following CCHFV infection were statistically analyzed: **p < 0.01 and ***p < 0.001. VEGF-A showed a trend of decrease that did not reach statistical significance.

All animals developed serum antibodies to CCHFV glycoprotein Gn and nucleoprotein as well as viral neutralizing activities (Results and discussion S1; Figure S3). Serum levels of 14 cytokines were quantified (Figures 3, S4 and S5). According to traditional classifications, IFN-γ, IP-10, IL-1α, MIP-1α, IL-8, VEGF-A, IL-1β, IL-17A, MIP-1β, and TNF-α are pro-inflammatory cytokines; IL-6 is typically regarded as a pro-inflammatory cytokine but has also demonstrated anti-inflammatory properties; and IL-10, IL-4, and IL-36Ra are anti-inflammatory cytokines.

In response to CCHFV infection, IFN-γ, IP-10, IL-6, and IL-10 exhibited significant increases largely coinciding with the viremic period followed by declines after peak viremia (Figure S4A–D). Increases were also found in IL-1α and MIP-1α, but with delayed kinetics extended into post-viremic time points (Figure S4E and F). A dramatic decrease in IL-8 was observed since 1 or 2 DPI, with the lowest IL-8 levels falling on time points around peak viremia. This was followed by a rebound that reached baseline levels by approximately 21 DPI (Figure 3A). A trend of similar decrease (in all animals) was found in VEGF-A, while it did not reach statistical significance (Figure 3B). Following an early trend of slight increase, IL-4 demonstrated a sharp drop after 3 or 4 DPI, with the lowest levels reached at time points shortly after peak viremia (7, 8, or 10 DPI) and followed by rebound later (Figure 3C). No changes consistent among all the animals were found in IL-1β, IL-17A, MIP-1β, TNF-α, or IL-36Ra (Figure S5A–E). A trend of IL-36Ra spike (in three of the four animals), however, was noticed at 14 DPI (Figure S5E), overlapping with the timing when the decreases in anti-Gn and anti-nucleoprotein antibodies as described above started to be observed (Figure S3A and B).

It has been proposed that CCHF pathogenesis in humans could be a result from massive, dysregulated inflammatory responses, possibly in combination with direct tissue injury by the virus, and intensely increased pro-inflammatory cytokines (cytokine storm) may mediate vascular dysfunction, DIC, organ failure and shock [32–35]. Out of a number of cytokines that have been assessed so far, increases in TNF-α, IL-8, IL-6, IL-1β, IP-10, VEGF-A, and IFN-γ were associated with poor outcomes [32,35–40]. Of those, TNF-α has been the most frequently reported predictive indicator of mortality. Recent studies have made progress toward understanding whether these correlations reflect causal, rather than purely coincidental, relationships. Blocking TNF-α signalling with TNF-α receptor knockout or a TNF-α neutralizing antibody afforded survival advantage in mouse models [15]. Furthermore, TNF-α derived from CCHFV-infected monocyte-derived dendritic cells mediated endothelial cell activation [41]. It was also known that TNF-α, IL-8, VEGF-A, IL-1β, and IL-4 increase endothelial permeability [42–54]. These are of significant mechanistic relevance as endothelial dysfunction and damage with leakage of erythrocytes and plasma through the vasculature into tissues is a hallmark of CCHF pathology. Endothelial damage contributes to coagulopathy by stimulating platelet aggregation and degranulation, with subsequent activation of the intrinsic coagulation cascade, leading to clotting factor deficiency and consequently haemorrhages [33,55].

In contrast to fatally infected humans, CCHFV-infected sheep were able to down-regulate these cytokines (IL-8, VEGF-A and IL-4; Figure 3) or limit their increase (TNF-α and IL-1β; Figure S5), which may serve as a defense mechanism against vascular dysfunction. The reduction of IL-8 in sheep may also be part of an inhibitory circuit restricting pathogenic recruitment of neutrophils as mentioned above. It should be noted that these possibilities remain to be tested in further experiments and other possibilities exist. Differential responses were similarly observed between interferon-α/β receptor knock-out mice and sheep. In these mice, lethal CCHFV infection exhibited significantly elevated levels of TNF-α, IL-1β, IL-4, and IL-17A [56], whereas these decreased or failed to increase in sheep. IL-17A was suggested in a previous study to synergize with TNF-α in neutrophil mobilization [57], which appeared to be limited in sheep.

The increases in other cytokines in CCHFV-infected sheep, along with the decreases or lack of significant changes in those discussed above, appeared to coincide with an induced anti-viral state with sharp reduction in viremia. Some increased cytokines may be part of an anti-viral response. Of these, IFN-γ was found to be critical for survival following CCHFV infection in interferon-α/β receptor knock-out mice [58]. Overall, the inflammatory cytokine responses were executed in sheep in a balanced manner as the effective control and resolution of viremia and the lack of remarkable inflammatory pathology were both achieved. The major changes in cytokine levels mostly corresponded to a limited window of acute viremia followed by their return to normal ranges. The timing, intensity and duration of these responses appeared to be within the ranges well tolerated by the host. As part of variations among individual animals, however, higher levels of some cytokines were noticed in sheep 21-04 including IL-1α, MIP-1α, IL-4, IL-1β, IL-17A, and TNF-α (Figures 3, S4 and S5). This was accompanied by a prolonged fever (Figure S1) and an extremely high level of viral RNA persistence in tissues (see below), while no major changes in the infection outcome were apparent. These results imply a complex nature of the biology underlying the lack of major disease, which was likely contributed to by the combination of multiple control mechanisms. Nevertheless, the immunological responses identified here especially those that differentiate between disease resistance and susceptibility host phenotypes provide promising targets for further mechanistic studies.

### Viral shedding, dissemination, and tissue persistence

**Figure 4.**
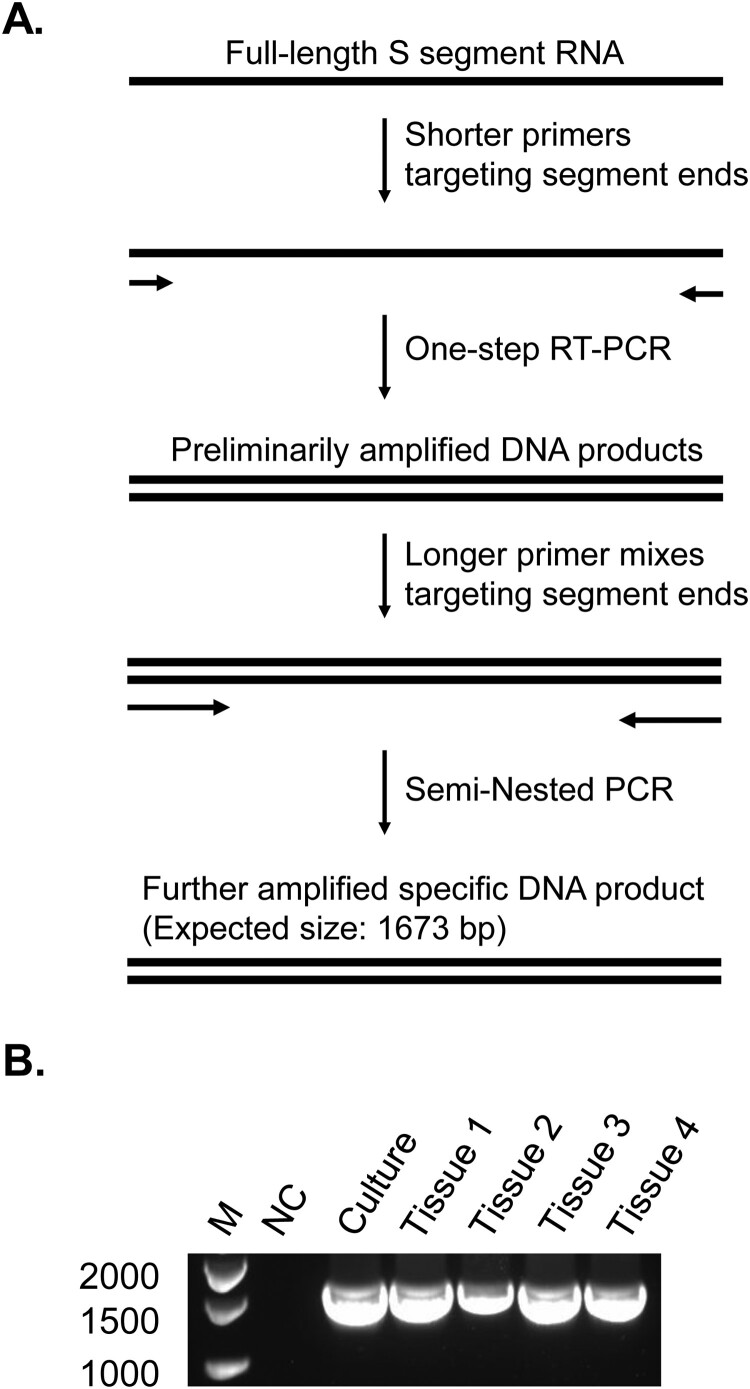
Long-term viral RNA persistence with the presence of full-length S segment in tissues of CCHFV-infected sheep. (A) Diagram (not drawn to scale) of a semi-nested PCR strategy to amplify the full-length CCHFV S segment in sheep tissues from 34 DPI. The primers of the second round PCR all retain the coverage of the extreme ends of the S segment, similarly to those of the first round PCR, but are longer with different 3’ sequences. A mixture of several such primers with various lengths was used to target each end of the template. bp, base pairs. Details of the PCR method including primer sequences are provided in Materials and Methods S1. (B) Agarose gel of PCR products. M, marker for DNA molecular sizes, with base pair numbers labelled on the left. NC, negative control without RNA template. Culture, supernatants of SW-13 cell culture infected with CCHFV. Tissue 1, Sheep 21-01 deep cervical lymph nodes. Tissue 2, Sheep 21-02 inguinal lymph nodes. Tissue 3, Sheep 21-03 inguinal lymph nodes. Tissue 4, Sheep 21-03 gastrohepatic lymph nodes. The bands were purified and sequenced and full-length S segment sequences were confirmed (Figure S6).

The virus spread beyond the blood in all animals. Nasal, oral, and rectal shedding of viral RNA was detected in swab elutes from several time points of the viremic period, ranging from 3 DPI to 6 DPI (Table S5). We analyzed tissues collected from necropsies on 34 DPI, the study end point, anticipating that no viral presence would be detected since the animals appeared to be able to clear the virus early based on the resolution of viremia by 6 DPI (Figure 1). To our surprise, however, viral RNA was detected extensively by RT-qPCR targeting part of the S segment in multiple types of lymph nodes (in all animals) as well as in the liver and spleen (in all animals except Sheep 21-04), and occasionally in other organ/tissue types including the ileum, adrenal gland, lung and potentially cerebrospinal fluid (Table 1). Among these, Sheep 21-04 and 21-01 demonstrated the highest viral RNA levels in inguinal lymph nodes and deep cervical lymph nodes, respectively. Furthermore, the presence of full-length S segment was demonstrated by conventional RT-PCR (Figure 4) and sequencing (Figure S6). These results indicate a widespread viral dissemination in the host and a long-term viral RNA persistence in tissues. Additionally, RT-PCR aimed at poly(A) or positive sense sequences of potential S segment transcripts generated positive amplifications (Figure S7). This appeared to suggest a possible presence of viral mRNA/positive-sense RNA in long-term tissues. Further studies should be carried out to address the presence of long term viral replication. It also remains to be determined whether functional virions are harboured or could be potentially produced in these tissues. Our virus isolation attempt, by applying tissue homogenates to SW-13 cell cultures, did not recover infectious virus. This could result from the limitation of the isolation conditions or the absence of readily packaged infectious virus.

**Table 1.** Viral RNA in tissues from 34 DPI.

Tissue type (ID number)	CCHFV RNA (10^3^ cp/ml)			
	Sheep 21-01	Sheep 21-02	Sheep 21-03	Sheep 21-04
Skeletal muscle: right quadriceps (1)	–/–	–/–	–/–	–/–
Inguinal lymph nodes (2)	**69.12**	**960.92**	**226.50**	**11082.04**
Gastrohepatic lymph nodes (3)	**49.77**	**250.00**	**988.83**	**49.28**
Liver (4)	**14.91**	**10.92**	**18.59**	–/–
Spleen (5)	**1.66**	**210.82**	**79.54**	–/–
Mesenteric lymph nodes (6)	**0.58/–**	–/–	**0.85**	–/–
Ileum (7)	–/–	–/–	–/–	**36.80**
Adrenal gland (8)	**0.61**	–/–	–/–	–/–
Kidney (9)	–/–	–/–	–/–	–/–
Lung: right middle lobe (10)	–/–	–/–	**4.48**	–/–
Heart (11)	–/–	–/–	–/–	–/–
Tracheobronchial lymph nodes (12)	**209.44**	**2.17**	**174.25**	**1.28**
Deep cervical lymph nodes (13)	**2514.43**	–/–	**5.11**	**101.36**
Cerebrum (14)	–/–	–/–	–/–	–/–
Cerebellum (15)	–/–	–/–	–/–	–/–
Hypothalamus (16)	–/–	–/–	–/–	–/–
Testicle (17)	–/–	–/–	–/–	–/–
Lung: left cranial lobe (18)	–/–	–/–	–/–	–/–
Additional lung tissue (19)	N/A	N/A	**27.61**	–/–
Cerebrospinal fluid (20)	–/–	–/–	**10.05/–**	–/–

CCHFV RNA in tissue homogenates was quantified by RT-qPCR. Mean value of duplicate samples is shown except “–/–” meaning both duplicate samples were negative in PCR and “#/–” meaning one of the duplicates positive while the other negative. Bold text indicates result with clinical significance. N/A, not applicable (tissue not tested).

Similar phenomena of viral RNA persistence after the clearance of acute infection have been emerging in other non-retroviral RNA viruses including Ebola virus, Marburg virus, Zika virus, and severe acute respiratory syndrome coronavirus 2 (SARS-CoV-2). These have been linked to recurring, chronic or progressive post-viral disease or syndromes and late transmissions of infectious virus that can spark new outbreaks [59,60]. It should be noted that regarding viral persistence for filoviruses the concept of persistence in immune privileged sites has been widely discussed [59]. However, the persistence of CCHFV RNA in sheep demonstrated localizations outside these sites (Table 1). The possible mechanism and impact of long-term CCHFV RNA persistence are discussed in the context of current literature (Results and discussion S1).

In conclusion, this study reveals previously unrecognized aspects of CCHFV biology in animals, presents a significant value of the CCHFV sheep infection model for studying host factors controlling outcomes of infection, for testing veterinary vaccines and for characterizing viral RNA persistence, and encourages and provides perspectives for extended future studies based on the revisit of experimental infection in animals (elaborated in Results and discussion S1).

## Supplementary Material

Supplementary_material_Corrected_1
