# Supplementary material for: Distinguishing host responses, extensive viral dissemination and long-term viral RNA persistence in domestic sheep experimentally infected with Crimean-Congo haemorrhagic fever virus Kosovo Hoti
Source: Emerg Microbes Infect. 2024 Jan 8;13(1):2302103. doi: 10.1080/22221751.2024.2302103 (PMC10810640; doi:10.1080/22221751.2024.2302103)
Supplement: Supplementary_material_Corrected_1 [file TEMI_A_2302103_SM4358.pdf]

Supplementary material

Distinguishing host responses, extensive viral dissemination and long-term viral RNA persistence in domestic sheep experimentally infected with Crimean-Congo haemorrhagic fever virus Kosovo Hoti

Hongzhao Li[1], Mathieu Pinette[1], Greg Smith[1], Melissa Goolia[1], Katherine Handel[1], Michelle Nebroski[1], Oliver Lung[1] and Bradley S Pickering[1,2*]

[1]National Centre for Foreign Animal Disease, Canadian Food Inspection Agency, Winnipeg, Manitoba, Canada;
[2]Department of Medical Microbiology and Infectious Diseases, College of Medicine, Faculty of Health Sciences, University of Manitoba, Winnipeg, Manitoba, Canada

[*] Correspondence to: bradley.pickering@inspection.gc.ca

Table of contents

Introduction S1……………………………………………………………………………...2
Materials and methods S1………….………………………………………………….......4
Results and discussion S1…………….………………………………………………......10
Table S1. Clinical findings and blood results in Sheep 21-01…………………….……….16
Table S2. Clinical findings and blood results in Sheep 21-02…………………….……….17
Table S3. Clinical findings and blood results in Sheep 21-03…………………….……….18
Table S4. Clinical findings and blood results in Sheep 21-04…………………….……….19
Table S5. Viral RNA shedding……………………………………………….……….20
Figure S1. Fever in CCHFV-infected sheep……………………...………………………...21
Figure S2. Blood chemistry changes following CCHFV infection..……………..………..…..22
Figure S3. Antibody responses in CCHFV-infected sheep.……………..…………………..…..23
Figure S4. Cytokines that demonstrated increases following CCHFV infection..……………..…24
Figure S5. Cytokines that did not show consistent and significant changes following CCHFV infection..…..………………………………………………………………………………25
Figure S6. Full-length S segment sequences identified in tissues from 34 DPI…………………26
Figure S7. RT-PCR aimed at poly(A) or positive sense sequences of potential S segment RNA transcripts in sheep tissues from 34 DPI…………….…………………………….…….…28
References……………………………………………………..………………...…………29

Introduction S1

CCHFV geographic expansion and recent trend of re-emergence

CCHFV has a widespread geographic distribution, involving Asia (from Western China to the Middle East and Turkey), Europe (Eastern and Southeastern countries) and Africa (majority of the continent). This vast geographic range reflects that tick hosts tolerate broadly diverse environments [1, 2]. There have been emerging signs that CCHFV is expanding into new territories such as Western Europe, which may be facilitated by emergence of favorable ecological environment driven by climate change and introduction of infected ticks by migratory birds or livestock trade [1-8]. In recent years there has been a trend of rising global incidence of CCHFV infections, with greatly increasing case numbers reported from several major endemic countries, demonstrating an imminent public health impact of this re-emerging virus [9-17].

Disease outcomes and prognostic markers of CCHFV infection in humans

In humans CCHFV infection can result in a range of disease outcomes [2, 18-20]. Most cases are asymptomatic or mild with non-specific symptoms, such as fever, headache, myalgia, dizziness, back and abdominal pains, nausea, vomiting and diarrhea. However, some cases quickly progress to a severe, often fatal, hemorrhagic fever disease (CCHF), characterized typically by vascular dysfunction, hemorrhagic manifestations, multi-organ failure (including cerebral, liver, and kidney failure and cardiac and pulmonary insufficiency), shock and death [2, 19-21]. Some CCHF survivors were reported to experience sequelae that may persist for longer than a year [18]. A number of laboratory findings are common correlates or predictors of poor outcomes, including high viral load (viremia), elevated serum levels of liver-associated enzymes, aspartate and alanine aminotransferases (AST and ALT), disseminated intravascular coagulation (DIC), thrombocytopenia, prolonged clotting times, absent or weak antibody response and increased serum levels of inflammatory cytokines and chemokines (both referred to as “cytokines” in this manuscript for simplicity) [2, 18-20, 22, 23].

CCHFV infection in animals

In wild and domestic vertebrate species, natural or experimental CCHFV infection does not appear to produce prominent disease, while a few exceptions exist in laboratory animals. Newborn mice, immunodeficient mice or hamsters, humanized mice and most recently cynomolgus macaques (with variable results) have demonstrated CCHF-like disease [2, 24-28]. For simplicity, other than these CCHF-susceptible laboratory animals, we refer to wild and domestic vertebrate species as “animals” hereafter. Field data on CCHFV infection in animals are primarily restricted to seroepidemiological surveys where CCHFV antibodies have been detected in a wide range of hosts, while the virus was isolated in a small number of cases [24, 29-34]. Like other large herbivores, domestic ruminants (sheep, cattle and goats) support and carry large numbers of infected ticks and exhibit a high prevalence of CCHFV antibodies [2, 35]. Sheep have been epidemiologically linked to human cases of CCHFV infection on a number of occasions [2, 35-40].

In experimental CCHFV infection studies, animals often develop a transient viremia, and viral transmission from infected animals to ticks have been observed in a number of cases. Birds do not develop detectable viremia, with a few exceptions; however, aviremic birds were reported to transmit the virus to ticks [41]. Due to the lack of obvious disease in animals, CCHFV is not

considered to have direct economic impact on livestock production. However, animals carrying viremia in addition to infected ticks represent a source of viral transmission. This has led to restrictive public health advices or measures, such as the ban of livestock transportation or slaughtering, or the closure of farms, which can cause economic impact indirectly [42-47].

Materials and Methods S1

Ethics and biosafety approval statement

All animal work was approved by the Animal Care Committee of the Canadian Science Centre for Human and Animal Health, under the animal user document number C-21-001. The study was designed and performed by strictly following the Canadian Council on Animal Care guidelines and Russell and Burch's 3Rs Principles [48, 49]. Animal housing, care, environmental enrichment and humane treatments during experimental procedures were conducted as previously described [50]. All invasive procedures, including viral inoculation, sample collection and euthanasia, were performed under isoflurane anesthesia. At the end of the study, animals were euthanized by intravenous administration of a commercial sodium pentobarbital solution. All experiments involving infectious CCHFV were conducted in the containment level 4 laboratory at the National Centre for Foreign Animal Diseases (NCFAD), Canadian Food Inspection Agency, following the institutional standard operating procedures.

Virus and cells

CCHFV Kosovo Hoti (also known as Kosova Hoti, GenBank accession numbers DQ133507, EU037902 and EU044832 for the S, M and L segments, respectively; passage 7) was acquired from the European Virus Archive – Global (https://www.european-virus-archive.com/virus/crimean-congo-hemorrhagic-fever-virus-strain-Kosovo-hoti; Ref-SKU: 007V-02504) [27, 51]. The original viral stock was subjected to one passage on SW-13 cells (ATCC, CCL-105) [52-54] to generate a working stock (passage 8) used for animal inoculation. The viral titer was determined on SW-13 cells using a TCID50 (50% tissue culture infectious dose) method. Detailed methods of viral production and titration were described previously, based on the CO_2^+ culture condition [54].

Viral load quantification by reverse transcription-quantitative polymerase chain reaction (RT-qPCR)

Sample inactivation was performed by mixing rigorously 175 µl sodium citrate-treated blood with 525 µl TRIzol LS Reagent (ThermoFisher Scientific, 10296028), or by mixing 70 µl swab elute or tissue homogenates with 630 µl TriPure Isolation Reagent (Sigma–Aldrich, 11667165001). Purification and RT-qPCR quantification of CCHFV RNA were conducted as previously described [54]. Briefly, the quantification targeted the S segment (nucleoprotein) using the primers CCHF-SF2 (GGAVTGGTGVAGGGARTTTG) and CCHF-SR2 (CADGGTGGRTTGAARGC) and the probe CCHF-N2 (6-FAM/CAARGGCAA/ZEN/RTACATMAT/IABkFQ). In vitro-transcribed S-segment RNA of CCHFV Kosovo Hoti was serially diluted and used as standards for quantification of viral RNA copy numbers.

Hematology, chemistry and coagulation times

Hematological analysis of blood cell counts and characteristics was performed using a VetScan HM5 hematology analyzer (Zoetis, https://www.zoetis.com/products-and-science/products/vetscan-hm5) on EDTA-treated whole blood. Blood chemistry including markers for liver and kidney diseases was analyzed on a VetScan VS2 chemistry analyzer (Zoetis, https://www.zoetis.com/products-and-science/products/vetscan-vs2) using the Preventative Care Profile Plus rotor with lithium heparin-treated whole blood. Citrated whole blood was used to

test for changes in coagulation times using Coag Dx Analyzer (IDEXX, https://www.idexx.com/en/veterinary/analyzers/coag-dx-analyzer/) with Citrated Blood PT (IDEXX, 99-13884) and Citrated Blood aPTT (IDEXX, 99-13885) cartridges. Compliance with the manufacturer's instructions was ensured for all the procedures. Details of the measured parameters and resulting data are provided in Tables S1-S4.

Indirect IgG ELISA

Nunc MaxiSorp flat bottom 96-well ELISA plates (Sigma-Aldrich, M9410) were coated with 50 ng/well of recombinant CCHFV Gn protein (Creative Diagnostics, DAGF-200) or nucleoprotein (Creative Diagnostics, DAGA-3109) in 50 µl 0.06 M carbonate/bicarbonate buffer with pH 9.6 at 4 °C overnight. Coated plates were washed three times using 0.01 M PBS containing 0.05% Tween20 with pH 7.2 (PBS-T). The wash was similarly carried out between each of the following steps. The plates were blocked with 5% skim milk in PBS-T (blocking buffer) for 1 hour (hr) at 37°C. Sera diluted at 1:500 in blocking buffer was added at 100 µl/ well and incubated at 37°C for 1 hr. The secondary antibody, Donkey anti-Sheep IgG (H+L) Secondary Antibody, HRP (ThermoFisher Scientific, A16041) was diluted at 1:2000 in blocking buffer and used at 100 µl/well with incubation at 37°C for 1 hr. For enzymatic color development, the Pierce TMB Substrate Kit (ThermoFisher Scientific, 34021) was used following the supplier's protocol. Optical density values were read on a BioTek Epoch Microplate Spectrophotometer (Agilent). In addition, similar ELISA methods were attempted using a recombinant CCHFV Gc protein (Creative Diagnostics, DAGA-3110), which generated false positive signals in the absence of CCHFV-infected sheep sera.

Virus neutralization test

Neutralizing antibody titers in serum samples were determined by a plaque reduction neutralization test (PRNT) against CCHFV. Serum samples were heat inactivated at 56 °C for 30 minutes (min), serially fivefold diluted and incubated with virus at 37 °C for 1 hr. Each serum-virus mixture was then applied to an antibody staining-based plaque assay described previously [54]. The highest dilution fold with > 70% reduction in plaque counts compared to uninfected control sera was defined as the neutralization titer.

Luminex cytokine assay

The MILLIPLEX Ovine Cytokine/Chemokine Panel 1 Premixed 14-plex - Immunology Multiplex Assay (Millipore Sigma, SCYT-91K-PX14) was used to quantify 14 cytokines in sheep serum samples following the manufacturer's instructions. These cytokines include interferon gamma (IFN-γ), interferon gamma-induced protein 10 (IP-10, also known as CXCL10), interleukin-1 alpha (IL-1α), macrophage inflammatory protein-1 alpha (MIP-1α, CCL3), IL-8 (CXCL8), vascular endothelial growth factor A (VEGF-A), IL-1β, IL-17A, MIP-1β (CCL4), tumor necrosis factor alpha (TNF-α), IL-6, IL-10, IL-4 and interleukin 36 receptor antagonist (IL-36Ra). Data were generated based on a 1:2 sample dilution for all cytokines except 1:8 for IL-8 and IP-10. The assay plate was run on a Luminex MagPix system using the xPONENT software of version 4.2.1324 (Luminex). Conversion of median fluorescent intensity (MFI) values to pg/ml was performed using an unweighted 5-parameter logistic method in xPONENT when all sample values for a cytokine fell within the calculation range of the standard curve.

Tissue homogenization

Tissues in Precellys bead mill tubes (Bertin, https://en.esbe.com) were mixed with DMEM (Wisent, 319-005-CL) at a 10% weight-to-total volume ratio and processed on a Minilys tissue homogenizer (Bertin, https://www.bertin-instruments.com) at maximum speed for 45 seconds. The homogenates were clarified as supernatants after centrifugation at 3000 g for 15 min (4 °C). For antibiotic treatment, homogenates were mixed with a 100× glutamine-antibiotic stock solution [55] at a 9:1 volume ratio (final antibiotic concentration at 10×) and incubated for 30 min at room temperature. The antibiotic-treated homogenates were further clarified by centrifugation as above. To make the 100× glutamine-antibiotic stock solution, a Solution A was prepared by dissolving 2.92 grams of L-glutamine (Sigma, G8540-25G) in 50 ml of sterile water and sterilized by passing through a 0.22 µm filter. A Solution B was prepared by dissolving 1,000,000 international units penicillin G sodium salt (Sigma, P3032-10MU), 1 gram streptomycin sulfate (Sigma, S9137-25G), 500,000 units nystatin (Sigma, N6261-500KU), 150,000 units polymyxin B sulfate (Sigma, P4932-1MU) and 1 gram active kanamycin monosulfate (Sigma, K1377-5G) in 5 to 10 ml of sterile water each and pooling these individual solutions together. Solution A and Solution B were then aseptically mixed and the total volume was brought to 100 ml with sterile water. The resulting 100× glutamine-antibiotic stock solution was aliquoted and stored at -20 °C.

Virus isolation

For virus isolation from blood, 100 µl/ well of sodium citrate-treated blood, undiluted or diluted at 1:100 or 1:1000 with DMEM (Wisent, 319-005-CL), was added onto SW-13 cells (approximately 90% confluent) cultured in 48 well plates following removal of old media. Viral adsorption was allowed for 1 hr. The inoculum was then removed and wells were washed two times with 300 µl maintenance media (DMEM + 2% FBS + 100 IU/ml penicillin and 100 µg/ml streptomycin), followed by addition of 600 µl fresh maintenance media. After incubation for 7 days, the growth of infectious CCHFV was determined by both the observation of cytopathic effect (CPE) in the cell culture [54] and the detection of viral RNA genome amplification with RT-qPCR as described above. It should be noted that no blood toxicity to SW-13 cells was observed; however, a 1:10 blood dilution in DMEM led to the formation of large clots, making the proper transfer of samples impossible. For virus isolation from tissues, untreated and antibiotic-treated tissue homogenates (described above), undiluted or diluted at 1:10 in DMEM, were subjected to the same virus isolation procedure as with blood. The exception, however, was that following the observation of no CPE on 5 DPI of the tissue viral isolation, the culture supernatants were transferred onto fresh SW-13 cells for a 7-day, second round of virus isolation test.

RT-PCR amplification of full-length S segment from tissue homogenates

A semi-nested PCR strategy was developed to address the molecular complexity in tissue homogenates and improve PCR specificity and the yield of full-length S amplicon. Total RNA extracted from tissue homogenates was preliminarily amplified using SuperScript™ III One-Step RT-PCR System with Platinum™ Taq DNA Polymerase (ThermoFisher Scientific, 12574018) and a pair of shorter primers targeting the ends of the S segment. These primers were fS1 (TCTCAAAGAAACACGTGCCGC) and rS1 (TCTCAAAGATATCGTTGCCGC), which were adapted for the Kosovo Hoti strain from previously published sequences [56]. The first round amplification reaction (50 µl) consisted of 16 µl water, 25 µl 2× Reaction Mix, 2 µl SuperScript

III RT/Platinum Taq Mix, 1 µl 10 mM primer fS1, 1 µl 10 mM primer rS1 and 5 µl RNA template. The thermal program was: RT at 52.5 °C for 30 minutes, pre-denaturation at 94 °C for 2 minutes, 40 amplification cycles (denaturation at 94 °C for 15 seconds, annealing at 50.5 °C for 30 seconds and extension at 68 °C for 1 minute 40 seconds), final extension at 68 °C for 5 minutes and cooling down at 4 °C for 5 minutes. The RT-PCR products were further amplified in the second round PCR, using longer primers which still covered the extreme ends of the S segment as the shorter primers of the first round PCR did but had different, extended 3' sequences. Each end of the S segment was targeted by a mix of several such primers with various lengths as follows:

Primer mix 1 included the following primers:
fS2 (TCTCAAAGAAACACGTGCCGCTTACG)
fS3 (TCTCAAAGAAACACGTGCCGCTTACGCCCA)
fS4 (TCTCAAAGAAACACGTGCCGCTTACGCCCACAGT)
fS5 (TCTCAAAGAAACACGTGCCGCTTACGCCCACAGTGTTCTC)
fS6 (TCTCAAAGAAACACGTGCCGCTTACGCCCACAGTGTTCTCTTGAGTGTCTG)

Primer mix 2 included the following primers:
rS2 (TCTCAAAGATATCGTTGCCGCACA)
rS3 (TCTCAAAGATATCGTTGCCGCACAGCCCT)
rS4 (TCTCAAAGATATCGTTGCCGCACAGCCCTTTAA)
rS5 (TCTCAAAGATATCGTTGCCGCACAGCCCTTTAAGTATTTG)
rS6 (TCTCAAAGATATCGTTGCCGCACAGCCCTTTAAGTATTTGAAATGAAAGATAG)

The second round PCR was conducted using LongAmp Taq PCR Kit (New England BioLabs, E5200S). The reaction (20 µl) consisted of 12 µl water, 4 µl 5× LongAmp Taq Reaction Buffer, 0.6 µl 10 mM dNTP mix, 0.8 µl 10 mM (total primer concentration) primer mix 1, 0.8 µl 10 mM (total primer concentration) primer mix 2, 0.8 µl LongAmp Taq and 1 µl first round PCR products. The thermal program was: pre-denaturation at 94 °C for 30 seconds, 40 amplification cycles (denaturation at 94 °C for 10 seconds, annealing and extension at 65 °C for 2 minutes 24 seconds), final extension at 65 °C for 10 minutes and cooling down at 4 °C for 5 minutes. The final PCR products were visualized on a 0.8% agarose gel with the molecular size marker 1 kb DNA Ladder (New England BioLabs, N3232S). DNA bands were purified using QIAquick Gel Extraction Kit (Qiagen, 28704) and sequenced as below.

Amplicon Sequencing

Gel-purified PCR amplicons for the S segment of CCHFV were submitted to NCFAD's Genomics Unit for high-throughput sequencing (HTS) and analysis. Samples were prepared using the Oxford Nanopore Technologies Rapid Barcoding 96 Kit (SQK-RBK110.96) according to the manufacturer's instructions (nanoporetech.com) with minor modifications on 200ng of each amplicon sample in nuclease-free water. The change was reducing the incubation time of the 1 µl unique rapid barcode and amplicon to 30^{0}C for 1 minute, followed by 80^{0}C for 1 minute, followed by a brief incubation on ice. The samples were pooled and then purified using the same volume of AMPure XP beads following Nanopore's protocol. The library was eluted in 11 µl of EB Buffer, then 1 µl of Rapid Adapter (RAP) was added. The library was sequenced on the

Nanopore GridION Mk1 instrument with an R9.4.1 Flow Cell (FLO-MIN106D, Oxford Nanopore Technologies) and base calling set to super-accurate with a 20bp read length cutoff.

Bioinformatics Analysis

Nanopore reads were trimmed for adapters using Porechop (v0.2.4) [57] on default settings. Following adapter trimming, quality trimming was performed with Chopper (v0.5.0) [58] on default settings except for the Q score which was set to 10 and the minimum read length, which was set to 200bp. Trimmed reads were mapped to the CCHFV reference (GenBank accession DQ133507.1) in Geneious Prime (2023.0.1)(https://www.geneious.com) with the Minimap2 assembler (v2.24) [59] on default settings to produce a 75% majority consensus sequence with the minimum consensus threshold set to 30x. The resulting consensus sequence was aligned to the reference sequence with MAFFT(v7.490) [60] in Geneious Prime (2023.0.1) to determine nucleotide pairwise identity.

RT-PCR aimed at poly(A) or positive sense sequences of potential S segment RNA transcripts in sheep tissues

RT was performed using Maxima H Minus First Strand cDNA Synthesis Kit (ThermoFisher Scientific, K1652). The RT primer was Oligo(dT)18 from the above kit or CCHF-SR2, the same primer as used in the RT-qPCR method described in the Materials and Methods section. The RNA template and RT primer were first subjected to denaturation and annealing in a reaction consisting of 8 µl water, 1 µl 100 µM RT primer, 5 µl sheep tissue RNA and 1 µl 10 mM dNTP mix, using the thermal program: 65 °C for 5 min and 4 °C for 5 min. This was followed by addition of 4 µl 5× RT buffer and 1 µl Maxima H Minus Enzyme Mix to complete a 20 µl reaction. The final RT thermal program was: 50 °C for 30 min (RT extension), 85°C for 5 min (RT enzyme inactivation) and 4 °C for 5 min. To purify cDNA, 10 µl RT reaction was digested with 1 µl RNase H (New England BioLabs, M0297L) and 1 µl RNase A from the RNaseAlert Lab Test Kit v2 (ThermoFisher Scientific, 4479768) at room temperature (21 °C) for 20 hr. cDNA was then purified using buffers from QIAquick Gel Extraction Kit (Qiagen, 28704) in combination with MinElute spin columns from MinElute Gel Extraction Kit (Qiagen, 28604), largely following the supplier's protocol for QIAquick Gel Extraction Kit. 5 µl RNase-digested RT reaction was mixed with 300 µl Buffer QG and 100 µl 2-propanol for loading onto a MinElute column. In the end, cDNA bound to the column was eluted with 10 µl water. Both rounds of nested PCR used LongAmp Taq PCR Kit (New England BioLabs, E5200S). For the first round PCR, the reaction consisted of 8 µl water, 4 µl 5× LongAmp Taq Reaction Buffer, 0.6 µl 10 mM dNTP mix, 0.8 µl 10 mM primer Nested1-F2-Hoti (TGGACACTTTCACAAACTC), 0.8 µl 10 mM primer Nested1-R3-Hoti (GACAAACTCCCTGCACCA), 0.8 µl LongAmp Taq and 5 µl purified cDNA template. The thermal program was: 94 °C for 30 seconds (s), 40 amplification cycles (94 °C for 10 s, 60 °C for 30 s and 65 °C 30 s), 65 °C for 10 min and 4 °C for 5min. For the second round PCR, the reaction consisted of 12 µl water, 4 µl 5× LongAmp Taq Reaction Buffer, 0.6 µl 10 mM dNTP mix, 0.8 µl 10 mM primer Nested2-F3-Hoti (GAATGTGCTTGGGTCAGCTC), 0.8 µl 10 mM primer Nested2-R2-Hoti (GACATCACAATTTCGCCAGG), 0.8 µl LongAmp Taq and 1 µl first round PCR reaction. The thermal program was: 94 °C for 30 s, 40 amplification cycles (94 °C for 10 s, 60 °C for 30 s and 65 °C 15 s), 65 °C for 10 min and 4 °C for 5 min. The final PCR products were visualized on a 2.5% agarose gel and the molecular size marker used was Invitrogen 100 bp DNA Ladder (ThermoFisher Scientific, 15628019). The four nested PCR primers described above were

adapted for the Kosovo Hoti strain from those commonly used in previous studies for CCHFV detection [42, 61, 62].

Statistical analysis

Significance of difference was determined by *Student's paired t* test between the baseline control group and infected group and by *Student's unpaired t* test between the enlarged NC group and infected group, using the GraphPad Prism software. A *p* value less than 0.05 was defined as significant.

Results and discussion S1

Discrepancy between viral RNA load and infectious virus, and high efficiency of CCHFV infectivity

As shown in Figure 1, the magnitude of viremia, or viral (RNA) load, did not always correlate with that of infectious virus. Three animals yielded successful virus isolation at the time points with peak viral load, whereas in Sheep 21-02 we were only able to isolate infectious virus from earlier time points with lower viral loads (2 DPI and 4 DPI), but not from the time point with peak viral load (6 DPI) (Figure 1). Similar discrepancy between the profiles of viral load and infectious virus titer was previously observed in CCHFV cell cultures [54]. The underlying mechanism is unknown, however this might be explained by the possibility that in some cases such as in Sheep 21-02, viral RNA could accumulate over time in the form of defective virus resulting from inactivation by the host immune response yet remaining to be cleared, or in the form of replication products including immature, partially assembled virions released from lysed host cells.

Nevertheless, it should be noted that CCHFV infectivity appeared to be highly efficient, even at viral RNA concentrations as low as a few hundred copies (cp)/ml blood. These were 552 and 3,458 cp/ml, respectively, on 2 DPI and 4 DPI in Sheep 21-02 (Figure 1). The isolation of the virus in the presence of a very low viremia should not be a result of contamination as none of the baseline samples tested alongside showed positive isolation. This was also the case for samples from other tested time points that were outside the viremic period, including 7 DPI and 8 DPI. Therefore, a low-level or potentially undetectable viremia does not necessarily indicate the lack of infectious virus and does not rule out the capability of the animal host to spread the virus. Consistent with our data, a previous study found that African ground-feeding birds, in the absence of detectable viremia, effectively transmitted CCHFV to ticks, and the virus was then successfully transmitted from ticks to rabbits [41].

Restriction of neutrophils as a potential mechanism for CCHF control in sheep

The role of neutrophils in the control or pathogenesis of CCHFV infection is poorly understood [63, 64]. Recent research has revealed a large functional versatility of neutrophils as mediators of immune response and inflammation including antiviral responses that limit viral replication and expansion, beyond their traditionally recognized role in simply killing invading bacteria. Neutrophils have also been implicated in pathological processes such as dysregulated inflammation, acute organ injury, and coagulation abnormalities [65]. Activated neutrophils produce pro-inflammatory cytokines such as IL-8, the prototype and most potent neutrophil-attracting and neutrophil-activating cytokine [66], and TNF-α, which can in turn activate more neutrophils and other immune cells [65, 67]. Studies suggested that a positive feedback loop of systemic and neutrophil autocrine IL-8 production and a feed-forward cascade involving neutrophils and pro-inflammatory cytokines (including TNF-α) contribute to the severity of COVID-19 and influenza diseases, respectively [68, 69]. With potential relevance to these findings, lethal CCHFV infection in interferon α/β receptor knockout mice was featured by neutrophil infiltrations found in the liver and spleen [64]. We hypothesize that neutrophils may exert a pathological effect on susceptible hosts toward fatal CCHF disease. Sheep may evade or counter such effect by restricting the recruitment of neutrophils into the blood stream. This appeared to occur as an early response to CCHFV infection, as observed on 1 DPI – 3 DPI

(Figure 2A and B). The restriction of recruitment may be through decreased mobilization of neutrophils from bone marrow into blood, increased storage or retention in the bone marrow or/and diminished production in the bone marrow [70]. Notably, the serum level of neutrophil chemoattractant IL-8 demonstrated a dramatic decrease (Figure 3) corresponding to the reduction of neutrophils in the blood.

Changes in blood biochemical markers for liver and kidney disease
A series of blood biochemical parameters (https://www.zoetis.es/_locale-assets/spc/rotor-vs2-comprehensive-diagnostic-profile.pdf) were utilized mainly as markers for potential disease in liver and kidney, which are typical targets by fatal CCHFV infection. Of the two most widely used liver disease markers for severe and fatal human CCHF, our analytical kit included ALT, without AST available. Significant changes were not found in albumin, alkaline phosphatase, ALT, bilirubin, calcium, phosphorus, creatinine, sodium, potassium, total protein or globulin (Tables S1-S4). These results suggest that there was no major injury to the liver or kidney. However, blood urea nitrogen (BUN) and calcium levels in all the animals demonstrated a consistent and significant decrease in early time windows of 1-2 DPI and 1-3 DPI, respectively, although with rebound and fluctuation at later time points (Figure S2A and B). In addition, all animals showed a trend of increase in blood glucose levels following infection, with subsequent decline or fluctuation (Figure S2C).

A major known cause of low BUN levels is liver disease [71-73]. Low blood calcium levels is a marker for kidney disease or worsening kidney function even when it is within the normal range or only mildly decreased [74, 75]. High blood glucose levels can result from increased hepatic glucose production and decreased renal glucose uptake caused by attenuated response to insulin in these organs under disease condition [76, 77]. Therefore, the changes in BUN, calcium and glucose levels together appeared to suggest a transient and minor impairment in certain biochemical functions of the liver and kidney. In CCHFV-infected African sheep, a slight but significant increase in AST was found, but no change in ALT was observed [78]. These results together with ours suggest that CCHFV infection in sheep may differentially affect some but not all disease markers since the effect of infection is not as major and extensive as in humans. However, the exact impact of CCHFV infection, especially potential long-term sequelae, on animal health status and production remains to be determined, as this area has never been covered by in-depth investigations, and variations in the biology caused by CCHFV infection among the animal population may have gone unrecognized.

Antibody responses
All animals developed antibodies in the serum to CCHFV glycoprotein Gn and nucleoprotein (Figure S3A and B). Anti-Gn IgG antibodies were first detected on 7 or 8 DPI and quickly spiked on 8 – 10 DPI. Interestingly, a decrease of antibody levels (as detected on 14 and 21 DPI) was followed by a rebound (28 and 34 DPI), which was consistently seen in all the animals (Figure S3A). This is reminiscent of the boost of a receding antibody response by a recurring antigen exposure. Anti-nucleoprotein IgG antibodies were initially detected on 4 or 5 DPI and demonstrated a gradual and continuous increase, except at late time points a trend of decrease followed by a rebound, similar to the observed pattern in anti-Gn antibodies (Figure S3B). The trend of decrease was perceivable at time points ranging from 10 to 21 DPI but to a lesser extent than in anti-Gn antibodies. The subsequent rebound, observed on 28 and 34 DPI, was

characterized by a further elevation above the levels prior to the decrease (Figure S3B). These findings imply a recurrence of CCHFV antigen long after the resolution of viremia, which appears to be consistent with a tissue persistence of CCHFV RNA as described later. Virus neutralization activity was detectable at nearly all time points on or after 6 DPI (Figure S3C). Together, the antibody data indicate that CCHFV-infected sheep mount a quick antibody response, in contrast to the lack of antibody response in fatal human cases or immunodeficient mice, which is a strong predictor of poor outcomes.

The glycoprotein Gc is the only known target of CCHFV-neutralizing antibodies [79]. Although antibodies to Gc were not measured in this study due to a lack of established method, the detection of virus neutralization activity (Figure S3C) represents an indirect/alternative evidence supporting that anti-Gc antibodies were likely developed in CCHFV-infected sheep. Currently, the role of anti-Gc/neutralizing antibodies in controlling CCHFV infection remains to be better understood. In immunodeficient mouse models, monoclonal neutralizing Gc-specific antibodies including those derived from convalescent donors were found to afford prophylactic protection but failed in more stringent therapeutic settings against established infection [79]. Characterization of neutralizing antibodies in immunocompetent hosts such as sheep should bring valuable new insights.

The possible mechanism and impact of long-term CCHFV RNA persistence

It remains unknown in what forms and by what mechanisms the viral RNA persists. A lack of culturable virus together with the known susceptibility of RNA to degradation points to the assumption that the viral sequences detected by RT-qPCR could be from fragmented RNA [80, 81]. However, dormant viral forms capable of reactivation, possibly with full-length RNA, have been suggested by the recrudescence of viral transcription and protein synthesis or infectious virus production in several viruses [80, 82-89]. Consistent with this, for CCHFV we detected full-length S segment in sheep tissues (Figure 4). It has been proposed that latent viral RNA may be protected in the cytoplasm of infected cells as ribonucleoprotein complexes or by association with membrane structures. Viral and host factors may suppress the production of infectious virions facilitating the survival of both the host cell and viral RNA against immune recognition with subsequent clearance [80]. Awakening from dormancy can occur when immune control is relaxed or in response to certain stimuli [80, 82-86].

Whether a similar dormant form of CCHFV persisted in the sheep is an open question. The antibody waves suggestive of recurring production of CCHFV protein antigens (Figure S3) appear to be consistent with this possibility. It should be noted that RNA persistence previously observed in other viruses was in the context of pathogenic infection, whereas CCHFV does not cause prominent disease in sheep and a widespread viral RNA persistence is much less anticipated. Thus, this unique finding is of great novelty and extends the known spectrum of types of infections with viral RNA persistence. Long-term health effects of CCHFV RNA persistence in sheep (notably related to the liver and the lymphatic system), however, should only be excluded by extended experimental studies, as these effects could potentially go unrecognized as seemingly non-specific variations in health status among the animal population. It must be noted that the highest levels of viral RNA persistence (Table 1) appeared to correlate with a prolonged fever and higher levels of inflammatory cytokines in Sheep 21-04 (Figures S1, 3, S4 and S5) and a late fever spike in Sheep 21-01 (Figure S1).

CCHFV RNA in the lymphoid organs may serve as sources of persistent antigenic stimulations directly within the immune system, as hinted by the anti-Gn and nucleoprotein antibody waves (Figure S3). Persistent immune activation has been implicated in immune exhaustion and increased re-infection by SARS-CoV-2 [81]. On the other hand, continued availability of antigenic boost is believed to be beneficial to the host for replenishing immunity such as in measles virus [90, 91]. These different possibilities warrant longer-term follow-up investigations in CCHFV-infected sheep.

It is noteworthy that windows of immune relaxation could allow the occasional release of infectious virus from latent reservoir reactivation as observed in Ebola, Zika and measles viruses, with relevance to late transmissions [85-89, 92-94]. Infectious virus potentially inducible from dormant CCHFV might be isolated with improved methods, which could be based on co-cultivation of tissues harboring persisting CCHFV RNA (as opposed to tissue homogenates) with susceptible cells, possibly in combination with immunosuppressive stimuli such as cyclophosphamide [85, 86, 92-94].

Epidemiological surveillance of CCHFV infections that have occurred in animals commonly depends on serological tests for CCHFV antibodies in conjunction with the ease of blood sampling. The use of the more sensitive and specific molecular methods, notably based on real-time PCR, has been limited by the consideration that viral RNA is only present in the blood for a short window during active replication. However, the findings of CCHFV RNA persistence suggest that PCR analysis of tissue RNA can be employed as an additional method to assess CCHFV infection in sheep (or possibly other animals to be found with CCHFV RNA persistence). Tissues collected during livestock slaughtering, for example, can serve as materials for testing. The PCR surveillance may complement serosurveys by catching cases corresponding to a waning phase of antibody responses, while it will provide information on the prevalence of CCHFV RNA persistence in animal populations.

Summary of updates on sheep susceptibility to CCHFV infection and its impact

CCHFV Kosovo Hoti-infected sheep developed a viremia in the absence of prominent clinical signs, confirming observations from past studies [24]. A cryptic CCHFV infection in livestock could pose great risk to public health due to unexpected transmission, especially in new regions where viral prevalence has not yet been noticed. In addition, although no major disease was manifested in infected animals, illness may be present but unrecognized. Markers for potential impairment in liver and kidney functions and viral RNA persistence in the liver, spleen, lymph nodes and some other types of organs/tissues, with a prolonged fever or late fever spike associated with high levels of viral RNA persistence, do advise possible impact of hidden CCHFV disease on animal health status and production levels, which has never been covered in experimental studies.

CCHFV infection in sheep as a promising model for addressing host factors that control disease, viral spread and viral RNA persistence

The differential outcomes of CCHFV infection in sheep and humans provide an opportunity for investigations into the host factors that control disease. Distinctive immune responses were identified in sheep that distinguish their subclinical infection from fatal infection in humans.

CCHFV-infected sheep were able to maintain and expand lymphocytes and develop quick antibody and cytokine responses associated with a rapid resolution of viremia. Notably, an early restriction of neutrophil recruitment and IL-8 levels may prevent pathogenic neutrophil infiltrations, and multiple cytokines with known roles in endothelial damage were found to be down-regulated or limited from increase, which may avoid vascular dysfunction and subsequent progression to severe disease. Genetic or pharmaceutical targeting of these distinguishing responses will determine whether they act as protective mechanisms against disease in sheep. Resulting knowledge will inform medical countermeasures that promote beneficial immune responses while limiting immunopathology.

The current knowledge regarding the host determinants of CCHFV infection outcomes has been hindered by the lack of ideal animal models. Studies have largely been limited to and focused on immunodeficient mice, as the field has been struggling to find an immunocompetent CCHF disease model. The mouse models have greatly contributed to our understanding of CCHFV pathogenesis but have limitations. These mice have incomplete and altered immune systems and type I interferon deficiency impacts both innate and adaptive immunity, which could lead to confounded findings or missing insights into natural responses that could otherwise be obtained in immunocompetent hosts. Indeed, the importance of lessons learned from natural immunity has been demonstrated by the advances from other viral disease models [95-97]. Although comparative studies in the context of immunocompetent hosts may be considered in human patients with different outcomes of CCHFV infection, in general they could not be conducted in-depth experimentally with major genetic or therapeutic manipulations. Instead, CCHFV infection in sheep can serve as a valuable immunocompetent animal model for studying host factors involved in pathogenesis and disease outcome, supplementing the immunodeficient mouse infection models.

This study brings pioneering findings of extensive CCHFV dissemination, viral shedding and viral RNA persistence in tissues in the context of immunocompetent animal hosts. Viral spread beyond the viremia presents additional sources of potential viral transmission, while this study has also confirmed the previously recognized role of blood based on past experimental data. Together, these findings support public health education and measures aimed to prevent or reduce the risk of acquiring CCHFV infection from infected animals, apart from infected ticks. Professionals at high risk such as farmers, slaughterhouse workers, veterinarians and stockmen should be made aware of these potential sources of infection from animals. Under the One Health approach, tick control has been implemented by the use of acaricides, which can be practically difficult under extensive farming conditions [43]. Alternatively, veterinary vaccines that block CCHFV replication hold promise to break animal host-mediated viral transmission chains to ticks and humans. In this regard, CCHFV Kosovo Hoti infection in sheep represents a valuable livestock model for the development of such vaccines, where vaccine effects on viremia and viral dissemination, shedding and tissue persistence can be tested. In addition, with CCHFV RNA long-term presence in sheep tissues this infection model carries an outstanding potential for studying viral RNA persistence in the context of disease-resistant hosts. While the potential health impact of CCHFV RNA persistence remains to be clarified, it may likely promote a durable anti-viral immunity.

Limitations of the study

Concerning the major limitation of this study, it was a pilot test of viral dissemination and persistence in tissues for CCHFV in animals and thus included only one late time point of animal sacrifice for tissue collection following the 3Rs animal use ethics. An enhanced and extended time course investigation with serial animal sacrifices will next determine the temporal and spatial sequence of viral dissemination through the organs/tissues in the host. The availability of frequent and extended tissue time points will also facilitate extensive characterization of viral RNA persistence, including the determination of time points and locations at which readily infectious virus or inducible virus or antigens could be isolated or detected. This will require a larger number of animals, which is now justifiable based on the new findings from the current study. In addition, the virus was inoculated through subcutaneous and intravenous injections, which might not faithfully represent the disease course after natural infection. Future studies should adopt a natural inoculation through tick bites and compare the outcomes based on these different routes of infection.

Table S1. Clinical findings and blood results in Sheep 21-01

				Baseline	Inoculation										
Time course	Date			2021-08-30	2021-08-31	2021-09-01	2021-09-02	2021-09-03	2021-09-04	2021-09-05	2021-09-06	2021-09-07	2021-09-08	2021-09-09	2021-09-10
	Days Post Infection (DPI)			-1	0	1	2	3	4	5	6	7	8	9	10
**Clinical signs**															
Temperature (°C)*	Rectal			39.4	39.6	39.4	41.5	40.4	39.9	41.3	41.1	40.4	39.9	39.8	39.7
Pulse oximetry				99%											
Heart rate	Beats per minute			160											
Attitude	0 = WNL, 1 = depressed			0	0	0	0	0	0	0	0	0	0	0	0
Appetite	0 = WNL, 1 = decreased			0	0	0	0	0	0	0	0	0	0	0	0
Diarrhea	0 = WNL, 1 = diarrhea			0	0	0	0	0	0	0	0	0	0	0	0
Other clinical				BAR	BAR; mild discharge from both eyes	BAR	QAR	QAR; some flushing around ears	QAR; mildly depressed	QAR; actively up and eating	BAR	BAR	BAR	BAR	BAR; dried serosanguinous nasal discharge bilateral
**VETSCAN HM5 Hematology Analyzer****	units	range low	range high												
WBC	10^9/l	4	12	7.69		8.07		6.38		7.44		10.42		11.94	
LYM	10^9/l	2	9	6.81		7.61		5.83		6.15		9.22		10.57	
MON	10^9/l	0	0.75	0.04		0.04		0.03		0.04		0.05		0.06	
NEU	10^9/l	0.7	6	0.85		0.42		0.52		1.25		1.15		1.32	
EOS	10^9/l	0		0.00		0.00		0.00		0.00		0.00		0.00	
BAS	10^9/l	0		0.00		0.00		0.00		0.00		0.00		0.00	
LYM %	%			88.5%		94.3%		91.4%		82.7%		88.5%		88.5%	
MON %	%			0.5%		0.5%		0.5%		0.5%		0.5%		0.5%	
NEU %	%			11.0%		5.2%		8.1%		16.8%		11.0%		11.0%	
EOS %	%			0.0%		0.0%		0.0%		0.0%		0.0%		0.0%	
BAS %	%			0.0%		0.0%		0.0%		0.0%		0.0%		0.0%	
RBC	10^{12}/l	9	15.8	13.00		12.59		12.72		12.60		12.95		12.37	
HGB	g/dl	9	15	13.6		13.8		13.6		14.0		14.4		13.3	
HCT	%	27	45	31.05%		30.97%		30.32%		29.78%		30.58%		29.34%	
MCV	fl	23	40	24		25		24		24		24		24	
MCH	pg	8	12	10.5		10.9		10.7		11.1		11.1		10.7	
MCHC	g/dl	31	47	43.9		44.5		44.8		46.9		47.0		45.2	
RDWc	%			31.3%		30.3%		31.3%		31.0%		31.0%		31.0%	
RDWs	fl			28.9		28.9		28.9		28.1		28.1		28.1	
PLT	10^9/l	100	800	299		316		522		523		783		469	
MPV	fl			5.8		6.1		5.8		5.8		5.9		5.8	
PCT	%			0.17%		0.19%		0.30%		0.31%		0.46%		0.27%	
PDWc	%			22.0%		23.5%		22.0%		22.0%		22.0%		22.0%	
PDWs	fl			5.3		5.9		5.3		5.3		5.3		5.3	
**VETSCAN VS2 Chemistry Analyzer****	units	range low	range high												
ALB	g/dl	2.4	3	2.9		3		2.7		2.7		2.7		2.6	
ALP	U/l	68	387	318		309		250		227		172		161	
ALT	U/l	26	34	14		13		15		16		13		16	
AMY	U/l	Not Used In Sheep		31		35		31		39		42		47	
TBIL	mg/dl	0.1	0.5	0.3		0.2		0.1		0.3		0.3		0.2	
BUN	mg/dl	8	20	15		12		16		15		19		11	
Ca++	mg/dl	11.5	12.8	9.8		10		9.6		10.5		10.4		10.4	
PHOS	mg/dl	5	7.3	6.9		8.1		6.3		6.3		6.4		7.1	
CRE	mg/dl	1.2	1.9	0.7		0.5		0.7		0.6		0.7		0.4	
GLU	mg/dl	50	80	70		73		74		80		89		74	
Na+	mEq/l	139	152	143		142		141		142		140		141	
K+	mEq/l	3.9	5.4	4.6		4.8		4.7		4.7		4.9		4.9	
TP	g/dl	6	7.9	6.4		6.5		6.7		6.6		7.1		6.9	
GLOB	g/dl	3.5	5.7	3.4		3.5		4		3.9		4.4		4.3	
HEM				0		0		0		0		0		0	
LIP				0		0		0		0		0		0	
ICT				0		0		0		0		0		0	
**IDEXX Coagulation Analyzer*****	units	range low	range high												
Citrate PT	seconds	26	38	44		45		39		41		37		38	
Citrate aPTT	seconds	64	96	88		83		86		88		92		71	

Time course	2021-09-11	2021-09-12	2021-09-13	2021-09-14	2021-09-15	2021-09-16	2021-09-17	2021-09-18	2021-09-19	2021-09-20	2021-09-21	2021-09-22
Days Post Infection (DPI)	11	12	13	14	15	16	17	18	19	20	21	22
**Clinical signs**												
Temperature (°C)*	40.1	40.5	40.2	39.8	39.7	39.8	39.3	39.9	39.5	39.8	39.8	40.0
Pulse oximetry												
Heart rate												
Attitude	0	0	0	0	0	0	0	0	0	0	0	0
Appetite	0	0	0	0	0	0	0	0	0	0	0	0
Diarrhea	0	0	0	0	0	0	0	0	0	0	0	0
Other clinical	BAR	BAR	BAR	BAR	BAR	BAR	BAR	BAR	BAR	BAR	BAR	BAR
**VETSCAN HM5 Hematology Analyzer****												
WBC				10.34							9.74	
LYM				9.45							8.61	
MON				0.05							0.05	
NEU				0.84							1.07	
EOS				0.00							0.00	
BAS				0.00							0.00	
LYM %				91.4%							88.5%	
MON %				0.5%							0.5%	
NEU %				8.1%							11.0%	
EOS %				0.0%							0.0%	
BAS %				0.0%							0.0%	
RBC				12.18							11.63	
HGB				12.7							12.1	
HCT				26.69%							27.85%	
MCV				24							24	
MCH				10.4							10.5	
MCHC				44.1							43.6	
RDWc				33.9%							31.0%	
RDWs				29.7							28.1	
PLT				576							327	
MPV				4.9							4.9	
PCT				0.28%							0.16%	
PDWc				18.6%							18.6%	
PDWs				3.7							3.7	
**VETSCAN VS2 Chemistry Analyzer****												
ALB				2.6							2.8	
ALP				213							443	
ALT				12							18	
AMY				53							53	
TBIL				0.2							0.3	
BUN				14							13	
Ca++				10.8							10.5	
PHOS				7.7							7.3	
CRE				0.6							0.7	
GLU				67							69	
Na+				140							143	
K+				5							5.2	
TP				7.2							6.6	
GLOB				4.6							3.8	
HEM				0							0	
LIP				0							0	
ICT				0							0	
**IDEXX Coagulation Analyzer*****												
Citrate PT				37							42	
Citrate aPTT				41							91	

												Euthanasia
Time course	2021-09-23	2021-09-24	2021-09-25	2021-09-26	2021-09-27	2021-09-28	2021-09-29	2021-09-30	2021-10-01	2021-10-02	2021-10-03	2021-10-04
Days Post Infection (DPI)	23	24	25	26	27	28	29	30	31	32	33	34
**Clinical signs**												
Temperature (°C)*	39.3	39.8	39.6	39.6	39.8	41.1	41.7	40.6	40.8	39.6	39.4	39.8
Pulse oximetry												
Heart rate												
Attitude	0	0	0	0	0	0	0	0	0	0	0	0
Appetite	0	0	0	0	0	0	0	0	0	0	0	0
Diarrhea	0	0	0	0	0	0	0	0	0	0	0	0
Other clinical	BAR	BAR	BAR	BAR	BAR	BAR	BAR	BAR	BAR	BAR	BAR	BAR
**VETSCAN HM5 Hematology Analyzer****												
WBC						8.58						8.72
LYM						7.59						7.72
MON						0.04						0.04
NEU						0.95						0.96
EOS						0.00						0.00
BAS						0.00						0.00
LYM %						88.5%						88.5%
MON %						0.5%						0.5%
NEU %						11.0%						11.0%
EOS %						0.0%						0.0%
BAS %						0.0%						0.0%
RBC						11.48						13.08
HGB						11.7						13.5
HCT						27.70%						31.32%
MCV						24						24
MCH						10.2						10.3
MCHC						42.1						43.1
RDWc						29.6%						31.0%
RDWs						27.3						28.1
PLT						212						450
MPV						4.9						5.8
PCT						0.10%						0.26%
PDWc						18.6%						22.0%
PDWs						3.7						5.3
**VETSCAN VS2 Chemistry Analyzer****												
ALB						2.9						2.7
ALP						395						283
ALT						18						18
AMY						42						36
TBIL						0.3						0.2
BUN						13						12
Ca++						10.3						10.4
PHOS						5.6						8
CRE						0.7						0.4
GLU						63						92
Na+						142						144
K+						5						5.8
TP						6.8						7.6
GLOB						3.8						4.9
HEM						0						0
LIP						0						0
ICT						0						0
**IDEXX Coagulation Analyzer*****												
Citrate PT						37						46
Citrate aPTT						85						84

Abbreviations: WNL = within normal limits; BAR = bright, alert, responsive; QAR = quiet, alert, responsive; WBC = total white blood cell count; LYM = lymphocyte count; MON = monocyte count; NEU = neutrophil count; EOS = eosinophil count; BAS = basophil count; LYM % = LYM / WBC; MON% = MON / WBC; NEU% = NEU / WBC; EOS % = EOS / WBC; BAS% = BAS / WBC; RBC = red blood cell count; HGB = hemoglobin; HCT = hematocrit; MCV = mean corpuscular volume; MCH = mean corpuscular hemoglobin; MCHC = mean corpuscular hemoglobin concentration; RDWc = red cell distribution width, coefficient of variation; RDWs = red cell distribution width, standard deviation; PLT = platelet count; MPV = mean platelet volume; PCT = platelet crit; PDWc = platelet distribution width, coefficient of variation; PDWs = platelet distribution width, standard deviation; ALB = albumin; ALP = alkaline phosphatase; ALT = alanine aminotransferase; AMY = amylase; TBIL = total bilirubin; BUN = urea nitrogen; Ca++ = total calcium; PHOS = phosphorus; CRE = creatinine; GLU = glucose; Na+ = sodium; K+ = potassium; TP = total protein; GLOB = globulin; HEM = hemolysis; LIP = lipemia; ICT = icterus; PT = prothrombin time; aPTT = activated partial thromboplastin time

Baseline values (from -1 DPI): underlined. **Color coding for parameters beyond reference ranges (see below):** Magenta = above reference range; Blue = below reference range.

References for parameter ranges in sheep:

*Rectal temperatures: Merck veterinary manual (https://www.merckvetmanual.com/multimedia/table/normal-rectal-temperature-ranges) and Clawson, AB. 1928 (https://doi.org/10.1152/ajplegacy.1928.85.2.251).

**Hematology and chemistry parameters: Appendix III from Pugh, DG. Sheep and Goat Medicine. 2002.

***Coagulation times: Connell, JM. 2007 (doi: 10.1097/MAT.0b013e31802e192b, or PMID: 17413565).

Table S2. Clinical findings and blood results in Sheep 21-02

Time course	Date	2021-08-30 (Baseline)	2021-08-31 (Inoculation)	2021-09-01	2021-09-02	2021-09-03	2021-09-04	2021-09-05	2021-09-06	2021-09-07	2021-09-08	2021-09-09	2021-09-10	2021-09-11	2021-09-12	2021-09-13	2021-09-14	2021-09-15	2021-09-16	2021-09-17	2021-09-18	2021-09-19	2021-09-20	2021-09-21	2021-09-22	2021-09-23	2021-09-24	2021-09-25	2021-09-26	2021-09-27	2021-09-28	2021-09-29	2021-09-30	2021-10-01	2021-10-02	2021-10-03	2021-10-04 (Euthanasia)
	**Days Post Infection (DPI)**	**-1**	**0**	**1**	**2**	**3**	**4**	**5**	**6**	**7**	**8**	**9**	**10**	**11**	**12**	**13**	**14**	**15**	**16**	**17**	**18**	**19**	**20**	**21**	**22**	**23**	**24**	**25**	**26**	**27**	**28**	**29**	**30**	**31**	**32**	**33**	**34**
**Clinical signs**																																					
**Temperature (^{o}C)***	Rectal	39.4	39.3	39.6	40.1	40.6	41.0	41.0	41.1	40.4	39.6	39.5	39.7	39.2	39.6	39.8	39.5	39.4	39.7	39.3	39.9	40.2	39.6	39.9	39.8	39.6	39.4	39.3	39.4	39.6	39.6	39.9	39.5	39.5	39.4	39.5	39.4
**Pulse oximetry**		X																																			
**Heart rate**	Beats per minute	X																																			
**Attitude**	0 = WNL, 1 = depressed	0	0	0	0	0	0	0	0	0	0	0	0	0	0	0	0	0	0	0	0	0	0	0	0	0	0	0	0	0	0	0	0	0	0	0	0
**Appetite**	0 = WNL, 1 = decreased	0	0	0	0	0	0	0	0	0	0	0	0	0	0	0	0	0	0	0	0	0	0	0	0	0	0	0	0	0	0	0	0	0	0	0	0
**Diarrhea**	0 = WNL, 1 = diarrhea	0	0	0	0	0	0	0	0	0	0	0	0	0	0	0	0	0	0	0	0	0	0	0	0	0	0	0	0	0	0	0	0	0	0	0	0
**Other clinical**		BAR	BAR	BAR	QAR	QAR	QAR; slightly flushed, mildly depressed	QAR; actively up and eating	BAR	BAR	BAR	BAR	BAR; dried serosanguinous nasal discharge bilateral; ocular discharge from both eyes	BAR	BAR	BAR	BAR	BAR	BAR	BAR	BAR	BAR	BAR	BAR	BAR	BAR	BAR	BAR	BAR	BAR	BAR	BAR	BAR	BAR	BAR	BAR	BAR

Parameter	units	range low	range high	-1	2	4	6	8	10	14	21	28	34
**VETSCAN HM5 Hematology Analyzer****	units	range low	range high										
**WBC**	10^9/l	4	12	9.81	4.78	9.19	8.03	15.18	11.59	10.99	11.70	12.67	11.96
**LYM**	10^9/l	2	9	8.68	4.51	7.60	6.17	12.11	9.92	9.40	11.03	11.57	10.93
**MON**	10^9/l	0	0.75	0.05	0.02	0.05	0.04	0.08	0.06	0.05	0.06	0.06	0.06
**NEU**	10^9/l	0.7	6	1.08	0.25	1.55	1.82	3.00	1.61	1.53	0.61	1.03	0.97
**EOS**	10^9/l	0		0.00	0.00	0.00	0.00	0.00	0.00	0.00	0.00	0.00	0.00
**BAS**	10^9/l	0		0.00	0.00	0.00	0.00	0.00	0.00	0.00	0.00	0.00	0.00
**LYM %**	%			88.5%	94.3%	82.7%	76.9%	79.8%	85.6%	85.6%	94.3%	91.4%	91.4%
**MON %**	%			0.5%	0.5%	0.5%	0.5%	0.5%	0.5%	0.5%	0.5%	0.5%	0.5%
**NEU %**	%			11.0%	5.2%	16.8%	22.6%	19.7%	13.9%	13.9%	5.2%	8.1%	8.1%
**EOS %**	%			0.0%	0.0%	0.0%	0.0%	0.0%	0.0%	0.0%	0.0%	0.0%	0.0%
**BAS %**	%			0.0%	0.0%	0.0%	0.0%	0.0%	0.0%	0.0%	0.0%	0.0%	0.0%
**RBC**	10^{12}/l	9	15.8	12.50	13.44	13.85	13.64	13.47	13.30	12.25	11.96	12.35	11.59
**HGB**	g/dl	9	15	13.7	13.7	15.0	15.1	13.0	13.4	12.4	11.8	11.8	11.2
**HCT**	%	27	45	29.42%	31.36%	32.44%	31.86%	31.89%	30.91%	28.36%	28.10%	28.54%	25.83%
**MCV**	fl	23	40	24	23	23	23	24	23	23	23	23	22
**MCH**	pg	8	12	10.9	10.2	10.8	11.1	9.7	10.0	10.1	9.9	9.5	9.7
**MCHC**	g/dl	31	47	46.5	43.8	46.3	47.5	40.9	43.2	43.7	42.2	41.2	43.4
**RDWc**	%			27.2%	28.2%	28.2%	28.2%	27.6%	26.8%	26.8%	26.8%	26.8%	26.3%
**RDWs**	fl			24.2	24.2	24.2	24.2	25.0	23.4	23.4	23.4	23.4	22.7
**PLT**	10^9/l	100	800	679	614	747	422	316	507	612	467	492	367
**MPV**	fl			4.9	4.9	5.4	5.4	5.8	5.8	4.9	5.3	4.9	4.9
**PCT**	%			0.33%	0.30%	0.40%	0.23%	0.18%	0.29%	0.30%	0.25%	0.24%	0.18%
**PDWc**	%			18.6%	18.6%	19.5%	19.5%	22.0%	22.0%	18.6%	21.4%	18.6%	18.6%
**PDWs**	fl			3.7	3.7	4.4	4.4	5.3	5.3	3.7	4.7	3.7	3.7
**VETSCAN VS2 Chemistry Analyzer****	units	range low	range high										
**ALB**	g/dl	2.4	3	2.9	2.9	2.9	2.6	2.5	2.8	3.1	2.7	2.7	2.9
**ALP**	U/l	68	387	144	135	104	81	89	89	230	164	195	230
**ALT**	U/l	26	34	13	16	16	8	15	18	20	16	15	13
**AMY**	U/l	Not Used In Sheep		29	25	21	23	27	35	46	22	27	28
**TBIL**	mg/dl	0.1	0.5	0.3	0.3	0.3	0.3	0.2	0.3	0.3	0.1	0.1	0.1
**BUN**	mg/dl	8	20	20	18	16	16	15	13	14	14	15	19
**Ca++**	mg/dl	11.5	12.8	10.3	10	10.8	9.9	10.6	11.5	10.2	10.2	10.6	10.1
**PHOS**	mg/dl	5	7.3	8.1	6	6	4.8	5.4	6.4	8.5	7.5	7.2	8.3
**CRE**	mg/dl	1.2	1.9	0.8	0.7	0.8	0.8	0.6	0.4	0.6	0.4	0.6	0.4
**GLU**	mg/dl	50	80	76	85	68	71	67	78	69	76	78	73
**Na+**	mEq/l	139	152	145	142	139	141	143	145	144	144	142	147
**K+**	mEq/l	3.9	5.4	4.1	4.6	4.5	4.2	4.5	4.4	4.8	4.4	4.8	4.5
**TP**	g/dl	6	7.9	6.6	6.5	6.5	6.3	6.2	7.2	6	6.8	6.6	6.5
**GLOB**	g/dl	3.5	5.7	3.7	3.5	3.6	3.8	3.7	4.4	2.9	4.1	3.8	3.7
**HEM**				2	0	0	0	0	0	0	0	0	0
**LIP**				0	0	0	0	0	0	0	0	0	0
**ICT**				0	0	0	0	0	0	0	0	0	0
**IDEXX Coagulation Analyzer*****	units	range low	range high										
**Citrate PT**	seconds	26	38	40	41	45	40	47	41	44	40	38	36
**Citrate aPTT**	seconds	64	96	89	92	104	81	88	89	92	95	102	94

Abbreviations: WNL = within normal limits; BAR = bright, alert, responsive; QAR = quiet, alert, responsive; WBC = total white blood cell count; LYM = lymphocyte count; MON = monocyte count; NEU = neutrophil count; EOS = eosinophil count; BAS = basophil count; LYM % = LYM / WBC; MON% = MON / WBC; NEU% = NEU / WBC; EOS % = EOS / WBC; BAS% = BAS / WBC; RBC = red blood cell count; HGB = hemoglobin; HCT = hematocrit; MCV = mean corpuscular volume; MCH = mean corpuscular hemoglobin; MCHC = mean corpuscular hemoglobin concentration; RDWc = red cell distribution width, coefficient of variation; RDWs = red cell distribution width, standard deviation; PLT = platelet count; MPV = mean platelet volume; PCT = platelet crit; PDWc = platelet distribution width, coefficient of variation; PDWs = platelet distribution width, standard deviation; ALB = albumin; ALP = alkaline phosphatase; ALT = alanine aminotransferase; AMY = amylase; TBIL = total bilirubin; BUN = urea nitrogen; Ca++ = total calcium; PHOS = phosphorus; CRE = creatinine; GLU = glucose; Na+ = sodium; K+ = potassium; TP = total protein; GLOB = globulin; HEM = hemolysis; LIP = lipemia; ICT = icterus; PT = prothrombin time; aPTT = activated partial thromboplastin time

Baseline values (from -1 DPI): underlined. **Color coding for parameters beyond reference ranges (see below):** Magenta = above reference range; Blue = below reference range.

References for parameter ranges in sheep:

*Rectal temperatures: Merck veterinary manual (https://www.merckvetmanual.com/multimedia/table/normal-rectal-temperature-ranges) and Clawson, AB. 1928 (https://doi.org/10.1152/ajplegacy.1928.85.2.251).

**Hematology and chemistry parameters: Appendix III from Pugh, DG. Sheep and Goat Medicine. 2002.

***Coagulation times: Connell, JM. 2007 (doi: 10.1097/MAT.0b013e31802e192b, or PMID: 17413565).

Table S3. Clinical findings and blood results in Sheep 21-03

Time course	Date			2021-08-30 (Baseline)	2021-08-31 (Inoculation)	2021-09-01	2021-09-02	2021-09-03	2021-09-04	2021-09-05	2021-09-06	2021-09-07	2021-09-08	2021-09-09	2021-09-10	2021-09-11	2021-09-12	2021-09-13	2021-09-14	2021-09-15	2021-09-16	2021-09-17	2021-09-18	2021-09-19	2021-09-20	2021-09-21	2021-09-22	2021-09-23	2021-09-24	2021-09-25	2021-09-26	2021-09-27	2021-09-28	2021-09-29	2021-09-30	2021-10-01	2021-10-02	2021-10-03	2021-10-04 (Euthanasia)
	Days Post Infection (DPI)			-1	0	1	2	3	4	5	6	7	8	9	10	11	12	13	14	15	16	17	18	19	20	21	22	23	24	25	26	27	28	29	30	31	32	33	34
**Clinical signs**																																							
**Temperature (°C)***	Rectal			39.4	39.3	39.9	40.6	40.0	40.6	41.3	40.0	39.7	39.5	39.8	39.6	39.6	39.5	39.6	39.6	39.6	39.5	39.4	39.7	39.3	39.4	39.6	39.6	39.4	39.3	39.3	39.3	39.9	39.9	39.7	39.9	39.5	39.6	39.7	39.6
**Pulse oximetry**				98%																																			
**Heart rate**	Beats per minute			160																																			
**Attitude**	0 = WNL, 1 = depressed			0	0	0	0	0	0	0	0	0	0	0	0	0	0	0	0	0	0	0	0	0	0	0	0	0	0	0	0	0	0	0	0	0	0	0	0
**Appetite**	0 = WNL, 1 = decreased			0	0	0	0	0	0	0	0	0	0	0	0	0	0	0	0	0	0	0	0	0	0	0	0	0	0	0	0	0	0	0	0	0	0	0	0
**Diarrhea**	0 = WNL, 1 = diarrhea			0	0	0	0	0	0	0	0	0	0	0	0	0	0	0	0	0	0	0	0	0	0	0	0	0	0	0	0	0	0	0	0	0	0	0	0
**Other clinical**				BAR	BAR	BAR	QAR	QAR; flushed around ears, distal limbs	QAR; mildly depressed	QAR; actively up and eating	BAR	BAR	BAR	BAR	BAR; dried serosanguinous nasal discharge bilateral	BAR	BAR	BAR	BAR	BAR	BAR	BAR	BAR	BAR	BAR	BAR	BAR	BAR	BAR	BAR	BAR	BAR	BAR	BAR	BAR	BAR	BAR	BAR	BAR

Time course	Units	Range low	Range high	-1	0	1	2	3	4	5	6	7	8	9	10	11	12	13	14	15	16	17	18	19	20	21	22	23	24	25	26	27	28	29	30	31	32	33	34
**VETSCAN HM5 Hematology Analyzer****	units	range low	range high																																				
**WBC**	10^9/l	4	12	8.11		8.13		6.33		5.57		10.51		8.09					7.70							7.96							12.01						9.79
**LYM**	10^9/l	2	9	7.88		7.90		6.23		5.25		9.91		7.97					7.46							7.73							11.32						9.52
**MON**	10^9/l	0	0.75	0.04		0.04		0.03		0.03		0.05		0.04					0.04							0.04							0.06						0.05
**NEU**	10^9/l	0.7	6	0.19		0.19		0.06		0.29		0.55		0.08					0.18							0.18							0.63						0.23
**EOS**	10^9/l	0		0.00		0.00		0.00		0.00		0.00		0.00					0.00							0.00							0.00						0.00
**BAS**	10^9/l	0		0.00		0.00		0.00		0.00		0.00		0.00					0.00							0.00							0.00						0.00
**LYM %**	%			97.2%		91.2%		98.5%		94.3%		94.3%		98.5%					97.2%							97.2%							94.3%						97.2%
**MON %**	%			0.5%		0.5%		0.5%		0.5%		0.5%		0.5%					0.5%							0.5%							0.5%						0.5%
**NEU %**	%			2.3%		2.3%		1.0%		5.2%		5.2%		1.0%					2.3%							2.3%							5.2%						2.3%
**EOS %**	%			0.0%		0.0%		0.0%		0.0%		0.0%		0.0%					0.0%							0.0%							0.0%						0.0%
**BAS %**	%			0.0%		0.0%		0.0%		0.0%		0.0%		0.0%					0.0%							0.0%							0.0%						0.0%
**RBC**	10^{12}/l	9	15.8	11.27		11.18		11.63		11.41		11.15		10.75					10.87							11.28							10.95						11.18
**HGB**	g/dl	9	15	11.7		10.9		10.8		11.2		10.9		10.4					10.2							11.0							11.2						11.1
**HCT**	%	27	45	26.49%		26.06%		27.10%		26.63%		26.05%		25.18%					25.40%							26.99%							26.14%						26.26%
**MCV**	fl	23	40	24		23		23		23		23		23					23							24							24						23
**MCH**	pg	8	12	10.4		9.7		9.3		9.8		9.8		9.7					9.4							9.8							10.2						9.9
**MCHC**	g/dl	31	47	44.3		41.8		40.0		41.9		41.7		41.4					40.3							40.8							42.9						42.1
**RDWc**	%			30.7%		30.7%		30.7%		30.7%		30.3%		28.9%					31.0%							30.0%							29.6%						29.3%
**RDWs**	fl			27.3		27.3		27.3		27.3		26.6		25.8					28.1							28.1							27.3						26.6
**PLT**	10^9/l	100	800	277		353		268		245		248		197					247							366							314						380
**MPV**	fl			4.9		5.7		5.4		5.4		5.4		5.4					4.9							5.0							4.9						5.0
**PCT**	%			0.14%		0.20%		0.14%		0.13%		0.13%		0.11%					0.12%							0.18%							0.15%						0.19%
**PDWc**	%			18.6%		23.9%		19.5%		19.5%		19.5%		19.5%					18.6%							18.6%							18.6%						18.6%
**PDWs**	fl			3.7		5.6		4.4		4.4		4.4		4.4					3.7							3.7							3.7						3.7
**VETSCAN VS2 Chemistry Analyzer****	units	range low	range high																																				
**ALB**	g/dl	2.4	3	3		2.9		2.8		2.8		2.8		2.8					2.7							3.2							3						2.9
**ALP**	U/l	68	387	304		317		274		246		210		193					164							312							206						225
**ALT**	U/l	26	34	16		12		11		12		16		14					17							23							21						21
**AMY**	U/l	Not Used In Sheep		33		43		43		46		59		60					29							47							44						45
**TBIL**	mg/dl	0.1	0.5	0.3		0.2		0.2		0.2		0.1		0.1					0.1							0.2							0.2						0.3
**BUN**	mg/dl	8	20	20		16		18		20		20		15					16							14							16						19
**Ca++**	mg/dl	11.5	12.8	10.3		9.8		9.6		9.7		9.6		10.3					10.4							10.5							9.8						9.8
**PHOS**	mg/dl	5	7.3	7.3		8.3		8.2		8.5		8.5		8.7					8.6							9.4							7.9						6.6
**CRE**	mg/dl	1.2	1.9	0.8		0.7		0.6		0.6		0.8		0.4					0.5							0.7							0.6						0.8
**GLU**	mg/dl	50	80	81		79		81		84		78		78					72							74							75						67
**Na+**	mEq/l	139	152	140		143		141		142		144		143					142							144							143						141
**K+**	mEq/l	3.9	5.4	4.6		4.9		4.7		4.4		5		4.8					4.9							5.1							4.5						4.7
**TP**	g/dl	6	7.9	5.8		5.9		5.8		5.9		6.2		6.2					6.5							6.1							6.3						6.8
**GLOB**	g/dl	3.5	5.7	2.7		3		3		3.2		3.4		3.3					3.8							2.8							3.3						3.9
**HEM**				0		0		0		0		0		0					1							0							0						0
**LIP**				0		0		0		0		0		0					0							0							0						0
**ICT**				0		0		0		0		0		0					0							0							0						0
**IDEXX Coagulation Analyzer*****	units	range low	range high																																				
**Citrate PT**	seconds	26	38	66		47		41		42		49		43					45							48							42						50
**Citrate aPTT**	seconds	64	96	113		97		92		105		91		85					93							97							105						96

Abbreviations: WNL = within normal limits; BAR = bright, alert, responsive; QAR = quiet, alert, responsive; WBC = total white blood cell count; LYM = lymphocyte count; MON = monocyte count; NEU = neutrophil count; EOS = eosinophil count; BAS = basophil count; LYM % = LYM / WBC; MON% = MON / WBC; NEU% = NEU / WBC; EOS % = EOS / WBC; BAS% = BAS / WBC; RBC = red blood cell count; HGB = hemoglobin; HCT = hematocrit; MCV = mean corpuscular volume; MCH = mean corpuscular hemoglobin; MCHC = mean corpuscular hemoglobin concentration; RDWc = red cell distribution width, coefficient of variation; RDWs = red cell distribution width, standard deviation; PLT = platelet count; MPV = mean platelet volume; PCT = platelet crit; PDWc = platelet distribution width, coefficient of variation; PDWs = platelet distribution width, standard deviation; ALB = albumin; ALP = alkaline phosphatase; ALT = alanine aminotransferase; AMY = amylase; TBIL = total bilirubin; BUN = urea nitrogen; Ca++ = total calcium; PHOS = phosphorus; CRE = creatinine; GLU = glucose; Na+ = sodium; K+ = potassium; TP = total protein; GLOB = globulin; HEM = hemolysis; LIP = lipemia; ICT = icterus; PT = prothrombin time; aPTT = activated partial thromboplastin time

Baseline values (from -1 DPI): underlined. **Color coding for parameters beyond reference ranges (see below):** Magenta = above reference range; Blue = below reference range.

References for parameter ranges in sheep:
*Rectal temperatures: Merck veterinary manual (https://www.merckvetmanual.com/multimedia/table/normal-rectal-temperature-ranges) and Clawson, AB. 1928 (https://doi.org/10.1152/ajplegacy.1928.85.2.251).
**Hematology and chemistry parameters: Appendix III from Pugh, DG. Sheep and Goat Medicine. 2002.
***Coagulation times: Connell, JM. 2007 (doi: 10.1097/MAT.0b013e31802e192b, or PMID: 17413565).

Table S4. Clinical findings and blood results in Sheep 21-04

Time course		Baseline	Inoculation																
	Date	2021-08-30	2021-08-31	2021-09-01	2021-09-02	2021-09-03	2021-09-04	2021-09-05	2021-09-06	2021-09-07	2021-09-08	2021-09-09	2021-09-10	2021-09-11	2021-09-12	2021-09-13	2021-09-14	2021-09-15	2021-09-16
	Days Post Infection (DPI)	-1	0	1	2	3	4	5	6	7	8	9	10	11	12	13	14	15	16
Clinical signs																			
Temperature (°C)*	Rectal	39.7	39.1	39.9	40.3	40.4	40.4	41.3	40.5	40.0	40.0	40.1	40.0	40.2	40.2	40.6	40.5	40.4	40.7
Pulse oximetry		97%																	
Heart rate	Beats per minute	98																	
Attitude	0 = WNL, 1 = depressed	0	0	0	0	0	0	0	0	0	0	0	0	0	0	0	0	0	0
Appetite	0 = WNL, 1 = decreased	0	0	0	0	0	0	0	0	0	0	0	0	0	0	0	0	0	0
Diarrhea	0 = WNL, 1 = diarrhea	0	0	0	0	0	0	0	0	0	0	0	0	0	0	0	0	0	0
Other clinical		BAR	BAR; moderate discharge from both eyes	BAR	QAR	QAR	QAR; slightly flushed, mildly depressed	QAR; actively up and eating	BAR	BAR	BAR	BAR	BAR; dried serosanguinous nasal discharge bilateral; ocular discharge from both eyes	BAR	BAR	BAR	BAR	BAR	BAR

Time course																			Euthanasia
	Date	2021-09-17	2021-09-18	2021-09-19	2021-09-20	2021-09-21	2021-09-22	2021-09-23	2021-09-24	2021-09-25	2021-09-26	2021-09-27	2021-09-28	2021-09-29	2021-09-30	2021-10-01	2021-10-02	2021-10-03	2021-10-04
	Days Post Infection (DPI)	17	18	19	20	21	22	23	24	25	26	27	28	29	30	31	32	33	34
Clinical signs																			
Temperature (°C)*	Rectal	40.3	40.3	40.2	40.2	40.2	40.3	40.0	40.1	40.1	39.8	40.2	40.0	40.3	40.2	40.2	39.7	40.1	40.1
Pulse oximetry																			
Heart rate	Beats per minute																		
Attitude	0 = WNL, 1 = depressed	0	0	0	0	0	0	0	0	0	0	0	0	0	0	0	0	0	0
Appetite	0 = WNL, 1 = decreased	0	0	0	0	0	0	0	0	0	0	0	0	0	0	0	0	0	0
Diarrhea	0 = WNL, 1 = diarrhea	0	0	0	0	0	0	0	0	0	0	0	0	0	0	0	0	0	0
Other clinical		BAR	BAR	BAR	BAR	BAR	BAR	BAR	BAR	BAR	BAR	BAR	BAR	BAR	BAR	BAR	BAR	BAR	BAR

VETSCAN HM5 Hematology Analyzer**	units	range low	range high	-1	2	4	6	8	10	14	21	28	34
WBC	10^9/l	4	12	9.77	5.83	7.60	8.17	10.93	9.57	12.79	9.05	10.25	8.13
LYM	10^9/l	2	9	9.21	5.66	6.94	7.22	10.31	9.02	11.32	8.27	9.07	7.66
MON	10^9/l	0	0.75	0.05	0.03	0.04	0.04	0.05	0.05	0.06	0.05	0.05	0.04
NEU	10^9/l	0.7	6	0.51	0.13	0.62	0.90	0.57	0.50	1.41	0.73	1.13	0.42
EOS	10^9/l	0		0.00	0.00	0.00	0.00	0.00	0.00	0.00	0.00	0.00	0.00
BAS	10^9/l	0		0.00	0.00	0.00	0.00	0.00	0.00	0.00	0.00	0.00	0.00
LYM %	%			94.3%	97.2%	91.4%	88.5%	94.3%	94.3%	88.5%	91.4%	88.5%	94.3%
MON %	%			0.5%	0.5%	0.5%	0.5%	0.5%	0.5%	0.5%	0.5%	0.5%	0.5%
NEU %	%			5.2%	2.3%	8.1%	11.0%	5.2%	5.2%	11.0%	8.1%	11.0%	5.2%
EOS %	%			0.0%	0.0%	0.0%	0.0%	0.0%	0.0%	0.0%	0.0%	0.0%	0.0%
BAS %	%			0.0%	0.0%	0.0%	0.0%	0.0%	0.0%	0.0%	0.0%	0.0%	0.0%
RBC	10^{12}/l	9	15.8	10.83	11.27	11.95	12.02	11.75	10.83	10.97	12.20	11.33	11.17
HGB	g/dl	9	15	11.6	11.8	12.1	13.0	12.3	11.0	11.2	12.9	11.6	11.0
HCT	%	27	45	26.00%	27.49%	28.53%	28.87%	28.18%	26.32%	26.03%	29.16%	27.25%	26.89%
MCV	fl	23	40	24	24	24	24	24	24	24	24	24	24
MCH	pg	8	12	10.7	10.5	10.1	10.8	10.5	10.2	10.2	10.6	10.3	9.8
MCHC	g/dl	31	47	44.6	43.1	42.3	45.0	43.7	41.9	43.2	44.2	42.7	40.9
RDWc	%			27.9%	26.6%	27.9%	27.9%	27.9%	25.0%	27.9%	29.3%	27.9%	26.6%
RDWs	fl			25.8	25.0	25.8	25.8	25.8	23.4	25.8	26.6	25.8	25.0
PLT	10^9/l	100	800	210	130	257	272	181	148	399	697	328	384
MPV	fl			4.9	5.8	4.9	5.0	5.8	7.6	4.9	5.2	5.0	4.9
PCT	%			0.10%	0.08%	0.13%	0.14%	0.10%	0.11%	0.20%	0.37%	0.16%	0.19%
PDWc	%			18.6%	22.0%	18.6%	18.6%	22.0%	27.4%	18.6%	21.4%	18.6%	18.6%
PDWs	fl			3.7	5.3	3.7	3.7	5.3	8.4	3.7	4.7	3.7	3.7

VETSCAN VS2 Chemistry Analyzer**	units	range low	range high	-1	2	4	6	8	10	14	21	28	34
ALB	g/dl	2.4	3	2.8	2.8	2.7	2.6	2.8	2.8	2.9	2.8	2.9	2.9
ALP	U/l	68	387	244	265	225	221	211	180	217	250	253	345
ALT	U/l	26	34	11	15	10	11	11	12	14	14	17	20
AMY	U/l	Not Used In Sheep		33	24	21	56	49	56	39	28	27	23
TBIL	mg/dl	0.1	0.5	0.3	0.2	0.1	0.1	0.1	0.2	0.1	0.1	0.1	0.1
BUN	mg/dl	8	20	26	19	21	19	22	18	22	19	18	24
Ca++	mg/dl	11.5	12.8	9.6	8.9	10.2	9.3	10.2	10.4	9.7	10	10.2	9.7
PHOS	mg/dl	5	7.3	8.3	8.5	8	7.6	7.9	8.4	8.8	9.2	7.5	7.2
CRE	mg/dl	1.2	1.9	0.6	0.8	0.6	1	0.8	0.6	0.7	0.6	0.9	0.7
GLU	mg/dl	50	80	75	81	76	79	69	73	68	71	75	70
Na+	mEq/l	139	152	141	143	143	143	143	146	142	141	143	142
K+	mEq/l	3.9	5.4	4.7	4.8	4.9	4.9	5	5.1	4.6	5	5	4.8
TP	g/dl	6	7.9	6.2	6.2	6.3	6.3	6.8	6.9	7	7.4	7.1	6.6
GLOB	g/dl	3.5	5.7	3.1	3.5	3.6	3.7	4	4.1	4.1	4.6	4.2	3.7
HEM				0	0	0	0	0	0	0	0	0	0
LIP				0	0	0	0	0	0	0	0	0	0
ICT				0	0	0	0	0	0	0	0	0	0

IDEXX Coagulation Analyzer***	units	range low	range high	-1	2	4	6	8	10	14	21	28	34
Citrate PT	seconds	26	38	38	37	43	39	48	44	41	49	47	46
Citrate aPTT	seconds	64	96	70	71	95	84	85	81	95	96	99	91

Abbreviations: WNL = within normal limits; BAR = bright, alert, responsive; QAR = quiet, alert, responsive; WBC = total white blood cell count; LYM = lymphocyte count; MON = monocyte count; NEU = neutrophil count; EOS = eosinophil count; BAS = basophil count; LYM % = LYM / WBC; MON% = MON / WBC; NEU% = NEU / WBC; EOS % = EOS / WBC; BAS% = BAS / WBC; RBC = red blood cell count; HGB = hemoglobin; HCT = hematocrit; MCV = mean corpuscular volume; MCH = mean corpuscular hemoglobin; MCHC = mean corpuscular hemoglobin concentration; RDWc = red cell distribution width, coefficient of variation; RDWs = red cell distribution width, standard deviation; PLT = platelet count; MPV = mean platelet volume; PCT = platelet crit; PDWc = platelet distribution width, coefficient of variation; PDWs = platelet distribution width, standard deviation; ALB = albumin; ALP = alkaline phosphatase; ALT = alanine aminotransferase; AMY = amylase; TBIL = total bilirubin; BUN = urea nitrogen; Ca++ = total calcium; PHOS = phosphorus; CRE = creatinine; GLU = glucose; Na+ = sodium; K+ = potassium; TP = total protein; GLOB = globulin; HEM = hemolysis; LIP = lipemia; ICT = icterus; PT = prothrombin time; aPTT = activated partial thromboplastin time

Baseline values (from -1 DPI): underlined. **Color coding for parameters beyond reference ranges (see below):** Magenta = above reference range; Blue = below reference range.

References for parameter ranges in sheep:

*Rectal temperatures: Merck veterinary manual (https://www.merckvetmanual.com/multimedia/table/normal-rectal-temperature-ranges) and Clawson, AB. 1928 (https://doi.org/10.1152/ajplegacy.1928.85.2.251).

**Hematology and chemistry parameters: Appendix III from Pugh, DG. Sheep and Goat Medicine. 2002.

***Coagulation times: Connell, JM. 2007 (doi: 10.1097/MAT.0b013e31802e192b, or PMID: 17413565).

Table S5. Viral RNA shedding

Sheep ID	Shedding source	CCHFV RNA (10^3 cp/ml)														
		-1 DPI	1 DPI	2 DPI	3 DPI	4 DPI	5 DPI	6 DPI	7 DPI	8 DPI	9 DPI	10 DPI	14 DPI	21 DPI	28 DPI	34 DPI
21-/-01	Nasal	-/-	-/-		-/-		-/-		-/-		-/-		-/-	-/-	-/-	-/-
	Oral	-/-	-/-		-/-		-/-		-/-		-/-		-/-	-/-	-/-	-/-
	Rectal	-/-	-/-		**2.37**		**127.27**		-/-		-/-		-/-	-/-	-/-	-/-
21-/-02	Nasal	-/-		-/-		-/-		-/-		-/-		-/-	-/-	-/-	-/-	-/-
	Oral	-/-		-/-		-/-		**1.14**		-/-		-/-	-/-	-/-	-/-	-/-
	Rectal	-/-		-/-		-/-		**8.85**		-/-		-/-	-/-	-/-	-/-	-/-
21-/-03	Nasal	-/-	-/-		**41.81**		**78.12**		-/-		-/-		-/-	-/-	-/-	-/-
	Oral	-/-	-/-		-/-		**1.10**		-/-		-/-		-/-	-/-	-/-	-/-
	Rectal	-/-	-/-		-/-		**4.39/-**		-/-		-/-		-/-	-/-	-/-	-/-
21-/-04	Nasal	-/-		-/-		-/-		**3.16**		-/-		-/-	-/-	-/-	-/-	-/-
	Oral	-/-		-/-		**2.28**		**23.14**		-/-		-/-	-/-	-/-	-/-	-/-
	Rectal	-/-		-/-		-/-		-/-		-/-		-/-	-/-	-/-	-/-	-/-

CCHFV RNA in swab elutes was quantified by RT-qPCR. Mean value of duplicate samples is shown except "-/-" meaning both duplicate samples were negative in PCR and "#/-" meaning one of the duplicates was positive while the other negative. Bold text indicates result with clinical significance. Empty spot indicates that sample was not collected due to sampling on alternate days.

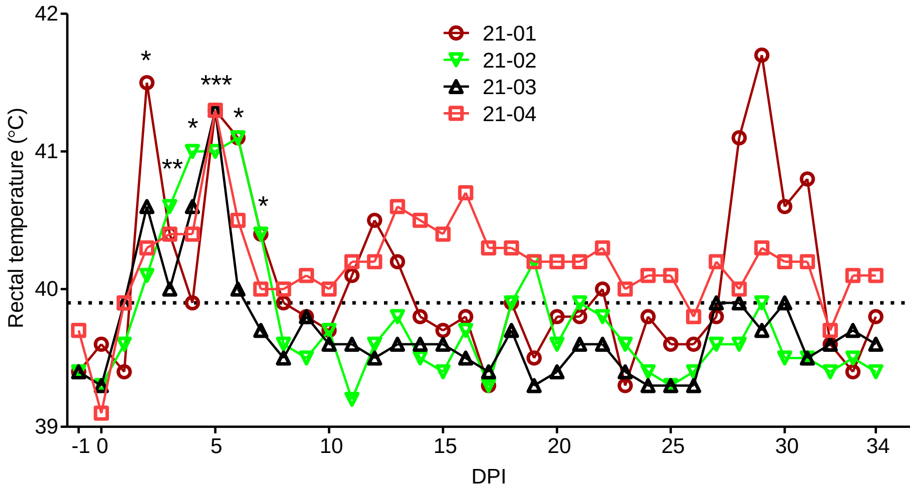

Figure S1. Fever in CCHFV-infected sheep. Line graph shows rectal temperature time course of each animal. Dash line indicates threshold temperature that defines a fever. Temperature significantly spiked following CCHFV infection as compared to the baseline (-1 DPI): *$p < 0.05$, **$p < 0.01$ and ***$p < 0.001$.

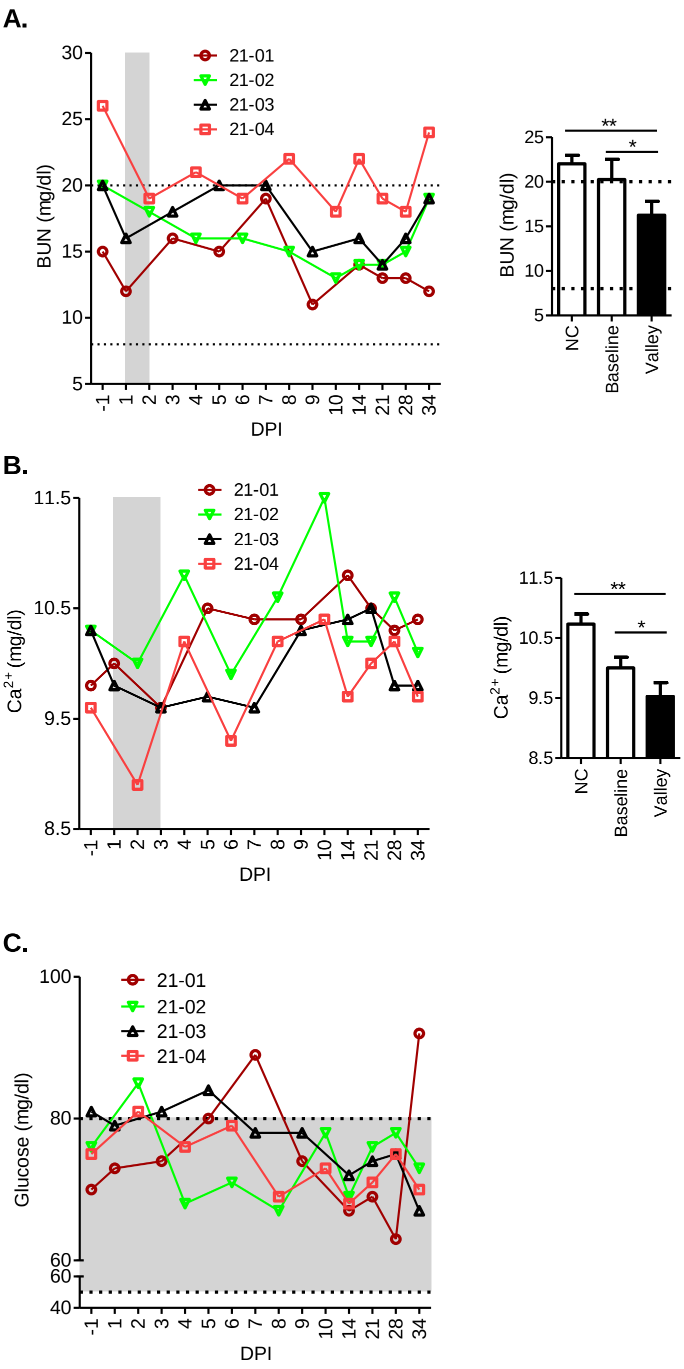

Figure S2. Blood chemistry changes following CCHFV infection. Data are presented in graph formats similar to those in Figure 2. BUN, blood urea nitrogen.

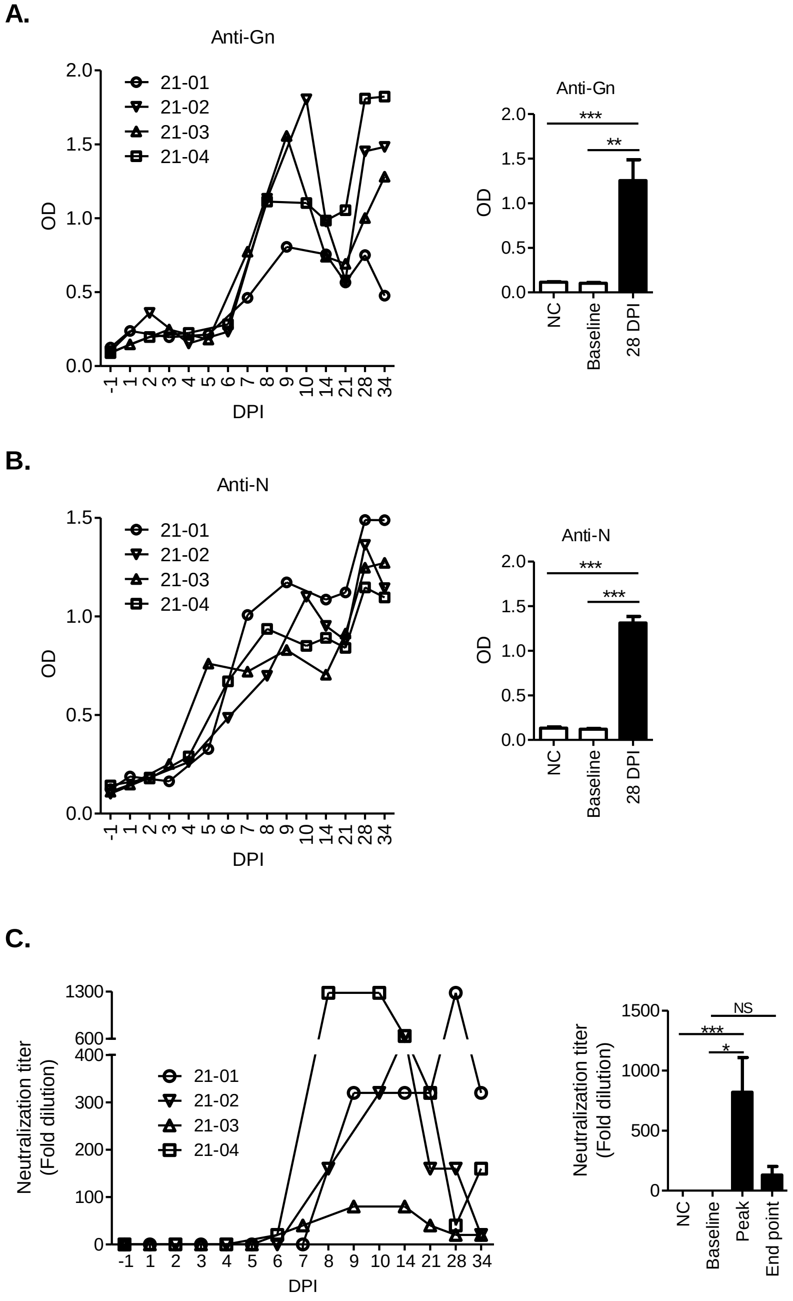

Figure S3. Antibody responses in CCHFV-infected sheep. A and B. Serum levels of anti-CCHFV Gn or nucleoprotein (N) IgG antibodies were measured by ELISA. OD, optical density. **C.** Neutralizing antibody titers in the serum were measured by virus neutralization test.

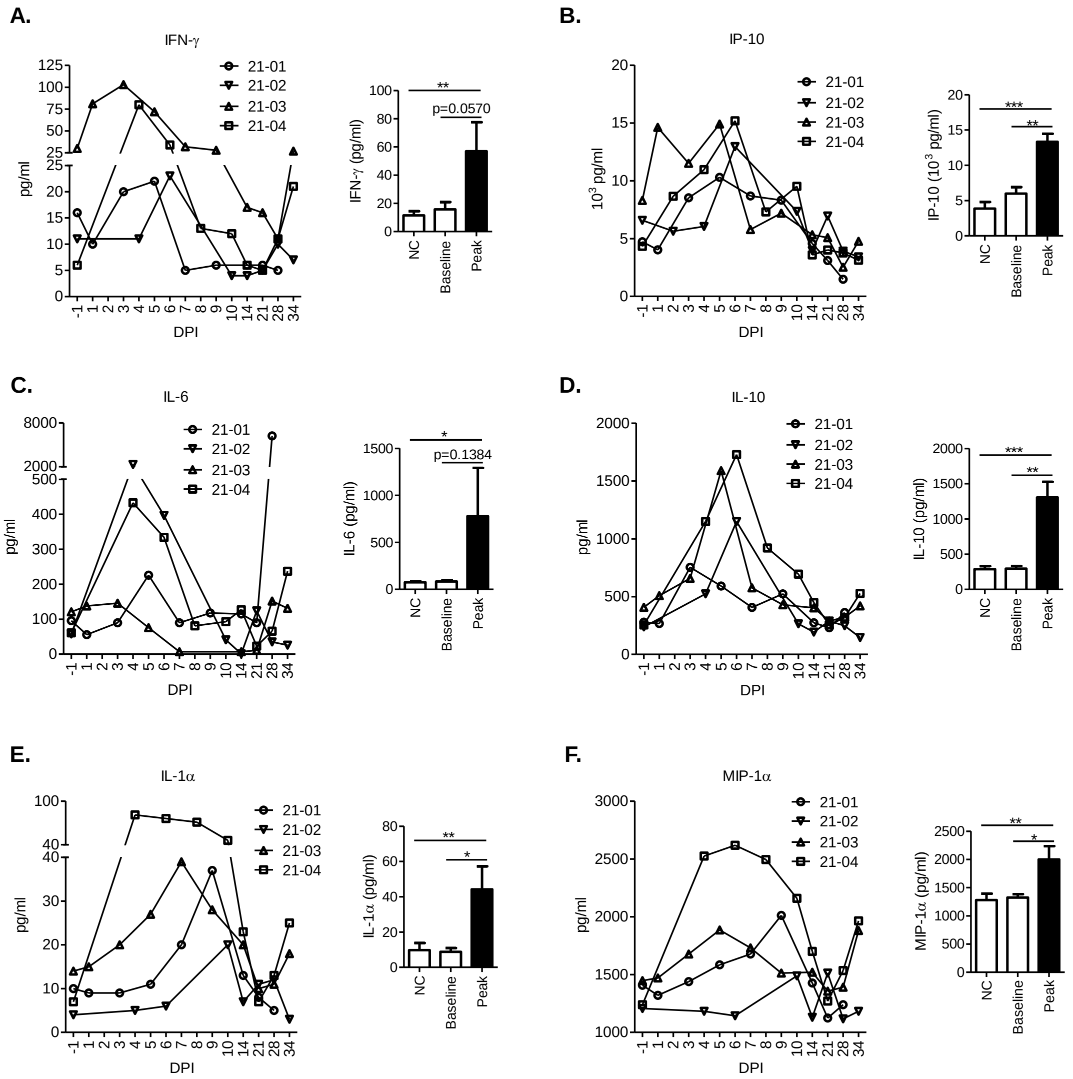

Figure S4. Cytokines that demonstrated increases following CCHFV infection. Serum levels of cytokines were measured by Luminex assay. Line graphs show the cytokine time course for each experimentally infected animal. Bar graphs (mean ± SEM) compare cytokine levels at their peak in the infected animals against those at the baseline (-1 DPI) or those in 14 uninfected, negative control (NC) animals. Increases in these cytokines following CCHFV infection were found statistically significant: *p < 0.05, **p < 0.01 and ***p < 0.001.

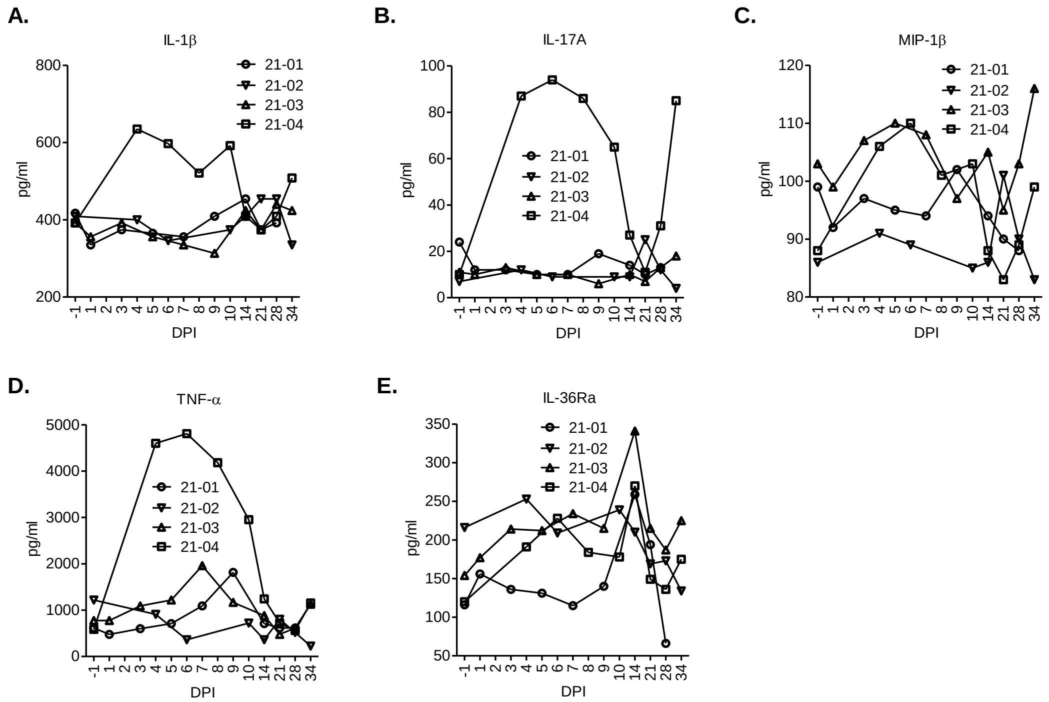

Figure S5. Cytokines that did not show consistent and significant changes following CCHFV infection. Serum levels of cytokines were measured by Luminex assay. Line graphs show the cytokine time course for each experimentally infected animal. No changes were found statistically significant.

```
Majority        XXXXXXXXAAACACGTGCCGCTTACGCCCACAGTGTTCTCTTGAGTGTCTGCAAAATGGAAAACAAGATCGAGGTGAACAGCAAAGATGAGATGAACAAA
                10 20 30 40 50 60 70 80 90 100
Reference.seq   TCTCAAAGAAACACGTGCCGCTTACGCCCACAGTGTTCTCTTGAGTGTCTGCAAAATGGAAAACAAGATCGAGGTGAACAGCAAAGATGAGATGAACAAA
Cellculture.seq NNNNNNNNNNNCACGTGCCGCTTACGCCCACAGTGTTCTCTTGAGTGTCTGCAAAATGGAAAACAAGATCGAGGTGAACAGCAAAGATGAGATGAACAAA
Tissue 1.seq    NNNNNNNNAAACACGTGCCGCTTACGCCCACAGTGTTCTCTTGAGTGTCTGCAAAATGGAAAACAAGATCGAGGTGAACAGCAAAGATGAGATGAACAAA
Tissue 2.seq    NNNNNNNNNAACACGTGCCGCTTACGCCCACAGTGTTCTCTTGAGTGTCTGCAAAATGGAAAACAAGATCGAGGTGAACAGCAAAGATGAGATGAACAAA
Tissue 3.seq    NNNNNNNNNAACACGTGCCGCTTACGCCCACAGTGTTCTCTTGAGTGTCTGCAAAATGGAAAACAAGATCGAGGTGAACAGCAAAGATGAGATGAACAAA
Tissue 4.seq    NNNNNNNNAAACACGTGCCGCTTACGCCCACAGTGTTCTCTTGAGTGTCTGCAAAATGGAAAACAAGATCGAGGTGAACAGCAAAGATGAGATGAACAAA

Majority        TGGTTTGAGGAGTTTAAAAAGGGAAATGGACTTATGGACACTTTCACAAACTCCTACTCCTTTTGCGAGAATGTACCAAATCTGGATAAGTTTGTGTTCC
                110 120 130 140 150 160 170 180 190 200
Reference.seq   TGGTTTGAGGAGTTTAAAAAGGGAAATGGACTTATGGACACTTTCACAAACTCCTACTCCTTTTGCGAGAATGTACCAAATCTGGATAAGTTTGTGTTCC
Cellculture.seq TGGTTTGAGGAGTTTAAAAAGGGAAATGGACTTATGGACACTTTCACAAACTCCTACTCCTTTTGCGAGAATGTACCAAATCTGGATAAGTTTGTGTTCC
Tissue 1.seq    TGGTTTGAGGAGTTTAAAAAGGGAAATGGACTTATGGACACTTTCACAAACTCCTACTCCTTTTGCGAGAATGTACCAAATCTGGATAAGTTTGTGTTCC
Tissue 2.seq    TGGTTTGAGGAGTTTAAAAAGGGAAATGGACTTATGGACACTTTCACAAACTCCTACTCCTTTTGCGAGAATGTACCAAATCTGGATAAGTTTGTGTTCC
Tissue 3.seq    TGGTTTGAGGAGTTTAAAAAGGGAAATGGACTTATGGACACTTTCACAAACTCCTACTCCTTTTGCGAGAATGTACCAAATCTGGATAAGTTTGTGTTCC
Tissue 4.seq    TGGTTTGAGGAGTTTAAAAAGGGAAATGGACTTATGGACACTTTCACAAACTCCTACTCCTTTTGCGAGAATGTACCAAATCTGGATAAGTTTGTGTTCC

Majority        AGATGGCCAGCGCCACTGATGATGCACAGAAGGACTCCATCTATGCATCGGCTCTGGTGGAAGCAACCAAGTTCTGTGCACCCATATATGAATGTGCTTG
                210 220 230 240 250 260 270 280 290 300
Reference.seq   AGATGGCCAGCGCCACTGATGATGCACAGAAGGACTCCATCTATGCATCGGCTCTGGTGGAAGCAACCAAGTTCTGTGCACCCATATATGAATGTGCTTG
Cellculture.seq AGATGGCCAGCGCCACTGATGATGCACAGAAGGACTCCATCTATGCATCGGCTCTGGTGGAAGCAACCAAGTTCTGTGCACCCATATATGAATGTGCTTG
Tissue 1.seq    AGATGGCCAGCGCCACTGATGATGCACAGAAGGACTCCATCTATGCATCGGCTCTGGTGGAAGCAACCAAGTTCTGTGCACCCATATATGAATGTGCTTG
Tissue 2.seq    AGATGGCCAGYGCCACTGATGATGCACAGAAGGACTCCATCTATGCATCGGCTCTGGTGGAAGCAACCAAGTTCTGTGCACCCATATATGAATGTGCTTG
Tissue 3.seq    AGATGGCCAGCGCCACTGATGATGCACAGAAGGACTCCATCTATGCATCGGCTCTGGTGGAAGCAACCAAGTTCTGTGCACCCATATATGAATGTGCTTG
Tissue 4.seq    AGATGGCCAGCGCCACTGATGATGCACAGAAGGACTCCATCTATGCATCGGCTCTGGTGGAAGCAACCAAGTTCTGTGCACCCATATATGAATGTGCTTG

Majority        GGTCAGCTCTACTGGCATTGTGAAGAAGGGGCTTGAGTGGTTTGAGAAGAATTCAGGAACCATCAAATCTTGGGATGAGAACTATGCTGAGCTGAAGGTT
                310 320 330 340 350 360 370 380 390 400
Reference.seq   GGTCAGCTCTACTGGCATTGTGAAGAAGGGGCTTGAGTGGTTTGAGAAGAATTCAGGAACCATCAAATCTTGGGATGAGAACTATGCTGAGCTGAAGGTT
Cellculture.seq GGTCAGCTCTACTGGCATTGTGAAGAAGGGGCTTGAGTGGTTTGAGAAGAATTCAGGAACCATCAAATCTTGGGATGAGAACTATGCTGAGCTGAAGGTT
Tissue 1.seq    GGTCAGCTCTACTGGCATTGTGAAGAAGGGGCTTGAGTGGTTTGAGAAGAATTCAGGAACCATCAAATCTTGGGATGAGAACTATGCTGAGCTGAAGGTT
Tissue 2.seq    GGTCAGCTCTACTGGCATTGTGAAGAAGGGGCTTGAGTGGTTTGAGAAGAATTCAGGAACCATCAAATCTTGGGATGAGAACTATGCTGAGCTGAAGGTT
Tissue 3.seq    GGTCAGCTCTACTGGCATTGTGAAGAAGGGGCTTGAGTGGTTTGAGAAGAATTCAGGAACCATCAAATCTTGGGATGAGAACTATGCTGAGCTGAAGGTT
Tissue 4.seq    GGTCAGCTCTACTGGCATTGTGAAGAAGGGGCTTGAGTGATTTGAGAAGAATTCAGGAACCATCAAATCTTGGGATGAGAACTATGCTGAGCTGAAGGTT

Majority        GATGTTCCCAAAATAGAACAACTTGCCAATTACCAACAGGCTGCTCTCAAGTGGAGGAAGGACATAGGTTTCCGTGTCAATGCAAACACGGCAGCCTTAA
                410 420 430 440 450 460 470 480 490 500
Reference.seq   GATGTTCCCAAAATAGAACAACTTGCCAATTACCAACAGGCTGCTCTCAAGTGGAGGAAGGACATAGGTTTCCGTGTCAATGCAAACACGGCAGCCTTAA
Cellculture.seq GATGTTCCCAAAATAGAACAACTTGCCAATTACCAACAGGCTGCTCTCAAGTGGAGGAAGGACATAGGTTTCCGTGTCAATGCAAACACGGCAGCCTTAA
Tissue 1.seq    GATGTTCCCAAAATAGAACAACTTGCCAATTACCAACAGGCTGCTCTCAAGTGGAGGAAGGACATAGGTTTCCGTGTCAATGCAAACACGGCAGCCTTAA
Tissue 2.seq    GATGTTCCCAAAATAGAACAACTTGCCAATTACCAACAGGCTGCTCTCAAGTGGAGGAAGGACATAGGTTTCCGTGTCAATGCAAACACGGCAGCCTTAA
Tissue 3.seq    GATGTTCCCAAAATAGAACAACTTGCCAATTACCAACAGGCTGCTCTCAAGTGGAGGAAGGACATAGGTTTCCGTGTCAATGCAAACACGGCAGCCTTAA
Tissue 4.seq    GATGTTCCCAAAATAGAACAACTTGCCAATTACCAACAGGCTGCTCTCAAGTGGAGGAAGGACATAGGTTTCCGTGTCAATGCAAACACGGCAGCCTTAA

Majority        GCAACAAAGTCCTTGCAGAATATAAAGTCCCTGGCGAAATTGTGATGTCTGTTAAAGAAATGCTGTCAGACATGATTAGAAGGAGGAATCTAATTCTCAA
                510 520 530 540 550 560 570 580 590 600
Reference.seq   GCAACAAAGTCCTTGCAGAATATAAAGTCCCTGGCGAAATTGTGATGTCTGTTAAAGAAATGCTGTCAGACATGATTAGAAGGAGGAATCTAATTCTCAA
Cellculture.seq GCAACAAAGTCCTTGCAGAATATAAAGTCCCTGGCGAAATTGTGATGTCTGTTAAAGAAATGCTGTCAGACATGATTAGAAGGAGGAATCTAATTCTCAA
Tissue 1.seq    GCAACAAAGTCCTTGCAGAATATAAAGTCCCTGGCGAAATTGTGATGTCTGTTAAAGAAATGCTGTCAGACATGATTAGAAGGAGGAAYCTAATTCTCAA
Tissue 2.seq    GCAACAAAGTCCTTGCAGAATATAAAGTCCCTGGCGAAATTGTGATGTCTGTTAAAGAAATGCTGTCAGACATGATTAGAAGGAGGAATCTAATTCTCAA
Tissue 3.seq    GCAACAAAGTCCTTGCAGAATATAAAGTCCCTGGCGAAATTGTGATGTCTGTTAAAGAAATGCTGTCAGACATGATTAGAAGGAGGAATCTAATTCTCAA
Tissue 4.seq    GCAACAAAGTCCTTGCAGAATATAAAGTCCCTGGCGAAATTGTGATGTCTGTTAAAGAAATGCTGTCAGACATGATTAGAAGGAGGAATCTAATTCTCAA

Majority        CAGGGGCGGTGATGAAAATCCACGCGGCCCAGTGAGCCGTGAACATGTGGAGTGGTGCAGGGAGTTTGTCAAAGGCAAGTACATCATGGCCTTCAATCCA
                610 620 630 640 650 660 670 680 690 700
Reference.seq   CAGGGGCGGTGATGAAAATCCACGCGGCCCAGTGAGCCGTGAACATGTGGAGTGGTGCAGGGAGTTTGTCAAAGGCAAGTACATCATGGCCTTCAATCCA
Cellculture.seq CAGGGGCGGTGATGAAAATCCACGCGGCCCAGTGAGCCGTGAACATGTGGAGTGGTGCAGGGAGTTTGTCAAAGGCAAGTACATCATGGCCTTCAATCCA
Tissue 1.seq    CAGGGGCGGTGATGAAAATCCACGCGGCCCAGTGAGCCGTGAACATGTGGAGTGGTGCAGGGAGTTTGTCAAAGGCAAGTACATCATGGCCTTCAATCCA
Tissue 2.seq    CAGGGGCGGTGATGAAAATCCACGCGGCCCAGTGAGCCGTGAACATGTGGAGTGGTGCAGGGAGTTTGTCAAAGGCAAGTACATCATGGCCTTCAATCCA
Tissue 3.seq    CAGGGGCGGTGATGAAAATCCACGCGGCCCAGTGAGCCGTGAACATGTGGAGTGGTGCAGGGAGTTTGTCAAAGGCAAGTACATCATGGCCTTCAATCCA
Tissue 4.seq    CAGGGGCGGTGATGAAAATCCACGCGGCCCAGTGAGCCGTGAACATGTGGAGTGGTGCAGGGAGTTTGTCAAAGGCAAGTACATCATGGCCTTCAATCCA

Majority        CCTTGGGGGGACATCAACAAATCAGGCCGTTCAGGAATAGCACTTGTTGCAACAGGCCTTGCCAAGCTTGCAGAGACCGAGGGGAAAGGAGTCTTTGACG
                710 720 730 740 750 760 770 780 790 800
Reference.seq   CCTTGGGGGGACATCAACAAATCAGGCCGTTCAGGAATAGCACTTGTTGCAACAGGCCTTGCCAAGCTTGCAGAGACCGAGGGGAAAGGAGTCTTTGACG
Cellculture.seq CCTTGGGGGGACATCAACAAATCAGGCCGTTCAGGAATAGCACTTGTTGCAACAGGCCTTGCCAAGCTTGCAGAGACCGAGGGGAAAGGAGTCTTTGACG
Tissue 1.seq    CCTTGGGGGGACATCAACAAATCAGGCCGTTCAGGAATAGCACTTGTTGCAACAGGCCTTGCCAAGCTTGCAGAGACCGAGGGGAAAGGAGTCTTTGACG
Tissue 2.seq    CCTTGGGGGGACATCAACAAATCAGGCCGTTCAGGAATAGCACTTGTTGCAACAGGCCTTGCCAAGCTTGCAGAGACCGAGGGGAAAGGAGTCTTTGACG
Tissue 3.seq    CCTTGGGGGGACATCAACAAATCAGGCCGTTCAGGAATAGCACTTGTTGCAACAGGCCTTGCCAAGCTTGCAGAGACCGAGGGGAAAGGAGTCTTTGACG
Tissue 4.seq    CCTTGGGGGGACATCAACAAATCAGGCCGTTCAGGAATAGCACTTGTTGCAACAGGCCTTGCCAAGCTTGCAGAGACCGAGGGGAAAGGAGTCTTTGACG

Majority        AAGCAAAGAAGACCGTGGAGGCTCTCAATGGGTATTTGGACAAGCACAGGGACGAAGTTGACAAAGCAAGTGCCGACAGCATGATAACAAACCTCCTAAA
                810 820 830 840 850 860 870 880 890 900
Reference.seq   AAGCAAAGAAGACCGTGGAGGCTCTCAATGGGTATTTGGACAAGCACAGGGACGAAGTTGACAAAGCAAGTGCCGACAGCATGATAACAAACCTCCTAAA
Cellculture.seq AAGCAAAGAAGACCGTGGAGGCTCTCAATGGGTATTTGGACAAGCACAGGGACGAAGTTGACAAAGCAAGTGCCGACAGCATGATAACAAACCTCCTAAA
Tissue 1.seq    AAGCAAAGAAGACCGTGGAGGCTCTCAATGGGTATTTGGACAAGCACAGGGACGAAGTTGACAAAGCAAGTGCCGACAGCATGATAACAAACCTCCTAAA
Tissue 2.seq    AAGCAAAGAAGACCGTGGAGGCTCTCAATGGGTATTTGGACAAGCACAGGGACGAAGTTGACAAAGCAAGTGCCGACAGCATGATAACAAACCTCCTAAA
Tissue 3.seq    AAGCAAAGAAGACCGTGGAGGCTCTCAATGGGTATTTGGACAAGCACAGGGACGAAGTTGACAAAGCAAGTGCCGACAGCATGATAACAAACCTCCTAAA
Tissue 4.seq    AAGCAAAGAAGACCGTGGAGGCTCTCAATGGGTATTTGGACAAGCACAGGGACGAAGTTGACAAAGCAAGTGCCGACAGCATGATAACAAACCTCCTAAA

Majority        GCACATTGCCAAAGCACAAGAGCTTTATAAAAATTCATCTGCTCTTCGTGCACAAGGTGCACAGATTGACACTCCTTTCAGCTCGTTTTACTGGCTCTAC
                910 920 930 940 950 960 970 980 990 1000
Reference.seq   GCACATTGCCAAAGCACAAGAGCTTTATAAAAATTCATCTGCTCTTCGTGCACAAGGTGCACAGATTGACACTCCTTTCAGCTCGTTTTACTGGCTCTAC
Cellculture.seq GCACATTGCCAAAGCACAAGAGCTTTATAAAAATTCATCTGCTCTTCGTGCACAAGGTGCACAGATTGACACTCCTTTCAGCTCGTTTTACTGGCTCTAC
Tissue 1.seq    GCACATTGCCAAAGCACAAGAGCTTTATAAAAATTCATCTGCTCTTCGTGCACAAGGTGCACAGATTGACACTCCTTTCAGCTCGTTTTACTGGCTCTAC
Tissue 2.seq    GCACATTGCCAAAGCACAAGAGCTTTATAAAAATTCATCTGCTCTTCGTGCACAAGGTGCACAGATTGACACTCCTTTCAGCTCGTTTTACTGGCTCTAC
Tissue 3.seq    GCACATTGCCAAAGCACAAGAGCTTTATAAAAATTCATCTGCTCTTCGTGCACAAGGTGCACAGATTGACACTCCTTTCAGCTCGTTTTACTGGCTCTAC
Tissue 4.seq    GCACATTGCCAAAGCACAAGAGCTTTATAAAAATTCATCTGCTCTTCGTGCACAAGGTGCACAGATTGACACTCCTTTCAGCTCGTTTTACTGGCTCTAC
```

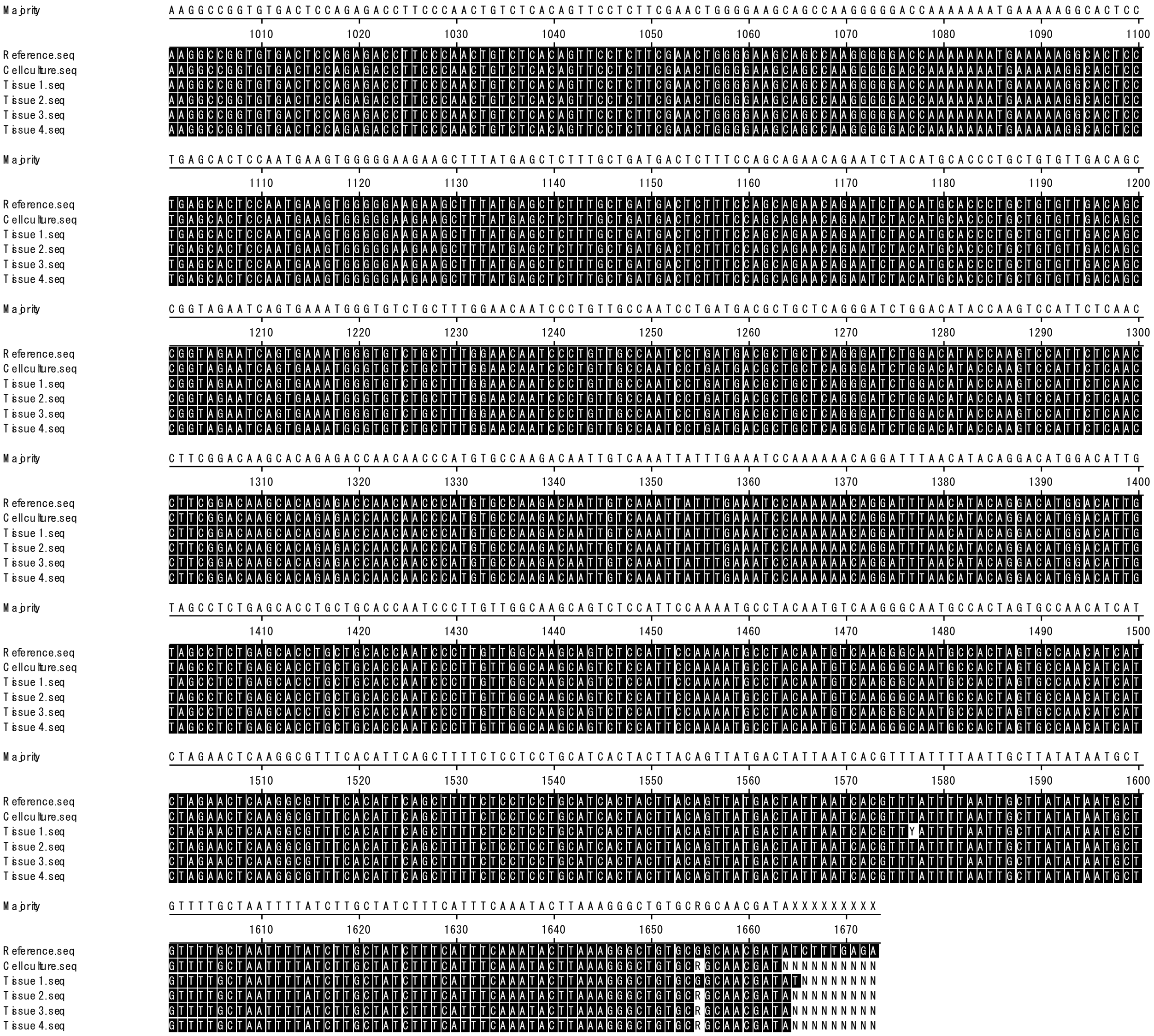

Figure S6. Full-length S segment sequences identified in tissues from 34 DPI. PCR amplicons from Figure 4 were gel-purified and subjected to Nanopore high-throughput sequencing. Sequences are shown in the positive sense format. Nucleotide residues are displayed with the standard codes by International Union of Pure and Applied Chemistry (IUPAC). Sequences were aligned by Clustal W method using DNASTAR Lasergene software. Reference, the CCHFV Kosovo Hoti reference sequence (GenBank accession number DQ133507.1). Cell culture, supernatants of SW-13 cell culture infected with CCHFV. Tissue 1, Sheep 21-01 deep cervical lymph nodes. Tissue 2, Sheep 21-02 inguinal lymph nodes. Tissue 3, Sheep 21-03 inguinal lymph nodes. Tissue 4, Sheep 21-03 gastrohepatic lymph nodes. Residues that match the reference are shaded. Note that this sequencing method is normally not expected to determine clearly the extreme ends (approximately 10 residues) of the linear templates. Such residues here are represented as “N”.

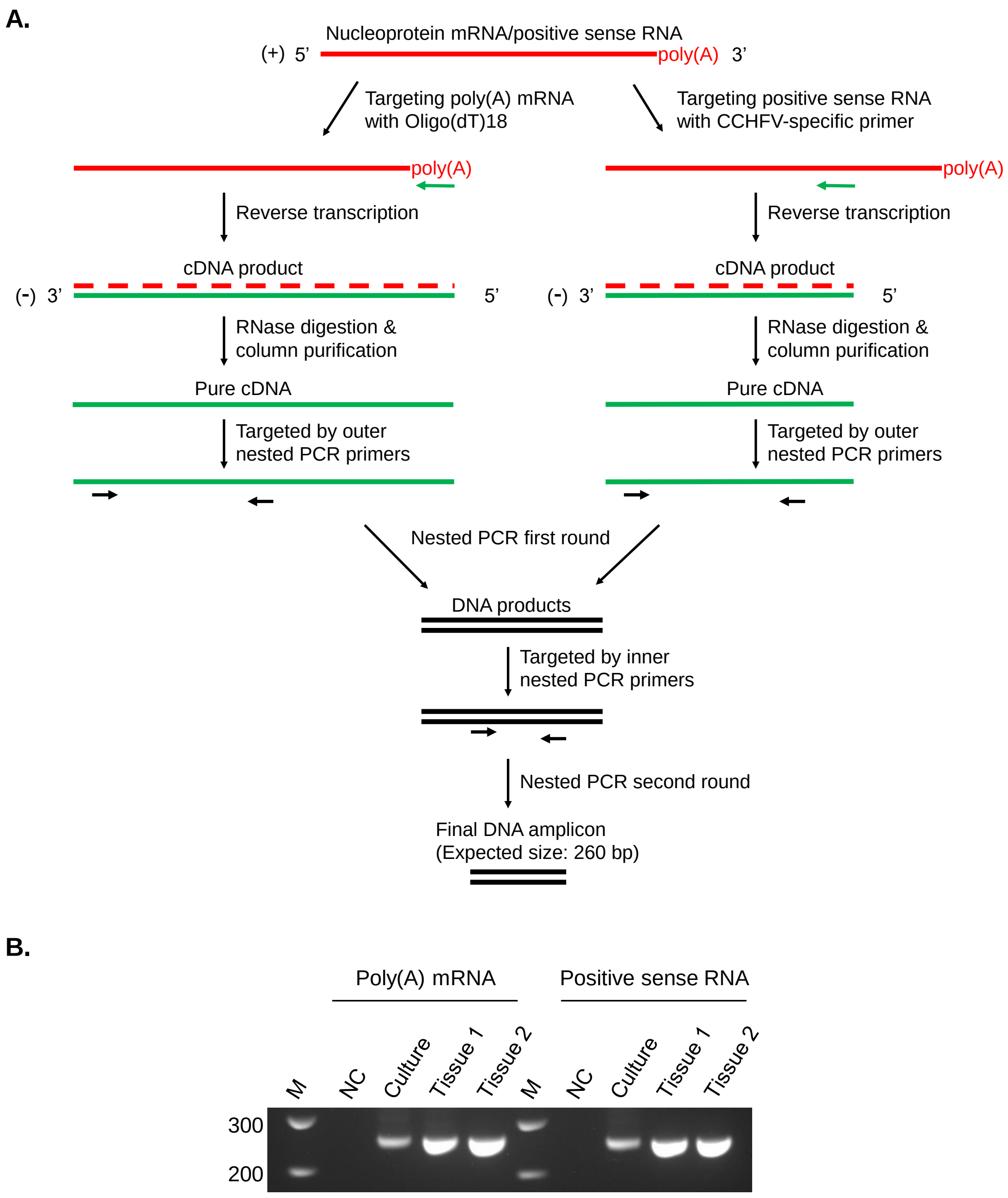

Figure S7. RT-PCR aimed at poly(A) or positive sense sequences of potential S segment RNA transcripts in sheep tissues from 34 DPI. A. Diagram (not drawn to scale) of a nested PCR strategy to detect poly(A) mRNA or positive sense transcripts. A single primer targeting poly(A) or internal CCHFV sequence of positive sense was used in a reverse transcription (RT) reaction. The reaction was then treated with RNases to remove RNA, including RNase H digestion of the hybrid RNA strand attached to cDNA and RNase A digestion of background RNA. This was followed by a column-based affinity purification of cDNA, which denatures proteins and specifically binds DNA. The purified cDNA was then subjected to nested PCR with two rounds of amplification using an outer pair and an inner pair of primers, respectively. Details of the RT and PCR methods are provided in Materials and Methods S1. **B.** Agarose gel of PCR products. M, marker for DNA molecular sizes, with base pair numbers labelled on the left. NC, negative control without RNA template. Culture, supernatants of SW-13 cell culture infected with CCHFV. Tissue 1, Sheep 21-01 deep cervical lymph nodes. Tissue 2, Sheep 21-02 inguinal lymph nodes.

References

1. Li H, Bello A, Smith G, Kielich DMS, Strong JE, Pickering BS. Degenerate sequence-based CRISPR diagnostic for Crimean-Congo hemorrhagic fever virus. PLoS Negl Trop Dis. 2022;16(3):e0010285.
2. Bente DA, Forrester NL, Watts DM, McAuley AJ, Whitehouse CA, Bray M. Crimean-Congo hemorrhagic fever: history, epidemiology, pathogenesis, clinical syndrome and genetic diversity. Antiviral Res. 2013;100(1):159-89.
3. Spengler JR, Bergeron E, Spiropoulou CF. Crimean-Congo hemorrhagic fever and expansion from endemic regions. Curr Opin Virol. 2019;34:70-8.
4. Mild M, Simon M, Albert J, Mirazimi A. Towards an understanding of the migration of Crimean-Congo hemorrhagic fever virus. J Gen Virol. 2010;91(Pt 1):199-207.
5. Grandi G, Chitimia-Dobler L, Choklikitumnuey P, Strube C, Springer A, Albihn A, et al. First records of adult Hyalomma marginatum and H. rufipes ticks (Acari: Ixodidae) in Sweden. Ticks Tick Borne Dis. 2020;11(3):101403.
6. Andersen LK, Davis MD. Climate change and the epidemiology of selected tick-borne and mosquito-borne diseases: update from the International Society of Dermatology Climate Change Task Force. Int J Dermatol. 2017;56(3):252-9.
7. Lorenzo Juanes HM, Carbonell C, Sendra BF, Lopez-Bernus A, Bahamonde A, Orfao A, et al. Crimean-Congo Hemorrhagic Fever, Spain, 2013-2021. Emerg Infect Dis. 2023;29(2):252-9.
8. Fanelli A, Schnitzler JC, De Nardi M, Donachie A, Capua I, Lanave G, et al. Epidemic intelligence data of Crimean-Congo haemorrhagic fever, European Region, 2012 to 2022: a new opportunity for risk mapping of neglected diseases. Euro Surveill. 2023;28(16).
9. Alhilfi RA, Khaleel HA, Raheem BM, Mahdi SG, Tabche C, Rawaf S. Large outbreak of Crimean-Congo haemorrhagic fever in Iraq, 2022. IJID Reg. 2023;6:76-9.
10. Tariq S, Niaz F, Safi Vahidy A, Qidwai M, Ishaq M, Abbasher Hussien Mohamed Ahmed K, et al. Crimean-Congo Hemorrhagic Fever (CCHF) in Pakistan: The Daunting Threat of an Outbreak as Eid-ul-Azha Approaches. Disaster Med Public Health Prep. 2023;17:e404.
11. Aslam M, Abbas RZ, Alsayeqh A. Distribution pattern of Crimean-Congo Hemorrhagic Fever in Asia and the Middle East. Front Public Health. 2023;11:1093817.
12. Fereidouni M, Apanaskevich DA, Pecor DB, Pshenichnaya NY, Abuova GN, Tishkova FH, et al. Crimean-Congo hemorrhagic fever virus in Central, Eastern, and South-eastern Asia. Virol Sin. 2023;38(2):171-83.
13. outbreaknewstoday.com. Afghanistan: Crimean-Congo hemorrhagic fever (CCHF) cases/deaths continue to rise. Available from: https://outbreaknewstoday.com/afghanistan-crimean-congo-hemorrhagic-fever-cchf-cases-deaths-continue-to-rise-79050/ [Cited 2023-08-03]. Outbreak News Today. 2023.
14. Shahhosseini N, Wong G, Babuadze G, Camp JV, Ergonul O, Kobinger GP, et al. Crimean-Congo Hemorrhagic Fever Virus in Asia, Africa and Europe. Microorganisms. 2021;9(9).
15. Blair PW, Kuhn JH, Pecor DB, Apanaskevich DA, Kortepeter MG, Cardile AP, et al. An Emerging Biothreat: Crimean-Congo Hemorrhagic Fever Virus in Southern and Western Asia. Am J Trop Med Hyg. 2019;100(1):16-23.
16. Leblebicioglu H, Ozaras R, Irmak H, Sencan I. Crimean-Congo hemorrhagic fever in Turkey: Current status and future challenges. Antiviral Res. 2016;126:21-34.
17. Keshtkar-Jahromi M, Sajadi MM, Ansari H, Mardani M, Holakouie-Naieni K. Crimean-Congo hemorrhagic fever in Iran. Antiviral Res. 2013;100(1):20-8.
18. Hoogstraal H. The epidemiology of tick-borne Crimean-Congo hemorrhagic fever in Asia, Europe, and Africa. J Med Entomol. 1979;15(4):307-417.
19. Whitehouse CA. Crimean-Congo hemorrhagic fever. Antiviral Res. 2004;64(3):145-60.
20. Ergonul O. Crimean-Congo haemorrhagic fever. Lancet Infect Dis. 2006;6(4):203-14.

21. Swanepoel R, Gill DE, Shepherd AJ, Leman PA, Mynhardt JH, Harvey S. The clinical pathology of Crimean-Congo hemorrhagic fever. Rev Infect Dis. 1989;11 Suppl 4:S794-800.
22. Ergonul O, Celikbas A, Baykam N, Eren S, Dokuzoguz B. Analysis of risk-factors among patients with Crimean-Congo haemorrhagic fever virus infection: severity criteria revisited. Clin Microbiol Infect. 2006;12(6):551-4.
23. Papa A, Tsergouli K, Caglayik DY, Bino S, Como N, Uyar Y, et al. Cytokines as biomarkers of Crimean-Congo hemorrhagic fever. J Med Virol. 2016;88(1):21-7.
24. Spengler JR, Estrada-Pena A, Garrison AR, Schmaljohn C, Spiropoulou CF, Bergeron E, et al. A chronological review of experimental infection studies of the role of wild animals and livestock in the maintenance and transmission of Crimean-Congo hemorrhagic fever virus. Antiviral Res. 2016;135:31-47.
25. Spengler JR, Kelly Keating M, McElroy AK, Zivcec M, Coleman-McCray JD, Harmon JR, et al. Crimean-Congo Hemorrhagic Fever in Humanized Mice Reveals Glial Cells as Primary Targets of Neurological Infection. J Infect Dis. 2017;216(11):1386-97.
26. Ranadheera C, Valcourt EJ, Warner BM, Poliquin G, Rosenke K, Frost K, et al. Characterization of a novel STAT 2 knock-out hamster model of Crimean-Congo hemorrhagic fever virus pathogenesis. Sci Rep. 2020;10(1):12378.
27. Haddock E, Feldmann F, Hawman DW, Zivcec M, Hanley PW, Saturday G, et al. A cynomolgus macaque model for Crimean-Congo haemorrhagic fever. Nat Microbiol. 2018;3(5):556-62.
28. Arnold CE, Shoemaker CJ, Smith DR, Douglas CE, Blancett CD, Graham AS, et al. Host response transcriptomic analysis of Crimean-Congo hemorrhagic fever pathogenesis in the cynomolgus macaque model. Sci Rep. 2021;11(1):19807.
29. Nurettin C, Engin B, Sukru T, Munir A, Zati V, Aykut O. The Seroprevalence of Crimean-Congo Hemorrhagic Fever in Wild and Domestic Animals: An Epidemiological Update for Domestic Animals and First Seroevidence in Wild Animals from Turkiye. Vet Sci. 2022;9(9).
30. Fanelli A, Buonavoglia D, Lanave G, Monaco F, Quaranta V, Catanzariti R, et al. First serological evidence of Crimean-Congo haemorrhagic fever virus in transhumant bovines in Italy. Transbound Emerg Dis. 2022;69(6):4022-7.
31. Mhamadi M, Badji A, Dieng I, Gaye A, Ndiaye EH, Ndiaye M, et al. Crimean-Congo Hemorrhagic Fever Virus Survey in Humans, Ticks, and Livestock in Agnam (Northeastern Senegal) from February 2021 to March 2022. Trop Med Infect Dis. 2022;7(10).
32. Dzikwi-Emennaa AA, Meseko C, Emennaa P, Adeyinka AJ, Adamu AM, Adegboye OA. Detection of Crimean-Congo Hemorrhagic Fever Virus Antibodies in Cattle in Plateau State, Nigeria. Viruses. 2022;14(12).
33. Atim SA, Niebel M, Ashraf S, Vudriko P, Odongo S, Balinandi S, et al. Prevalence of Crimean-Congo haemorrhagic fever in livestock following a confirmed human case in Lyantonde district, Uganda. Parasit Vectors. 2023;16(1):7.
34. Simo Tchetgna H, Yousseu FS, Cosset FL, de Freitas NB, Kamgang B, McCall PJ, et al. Molecular and serological evidence of Crimean-Congo hemorrhagic fever orthonairovirus prevalence in livestock and ticks in Cameroon. Front Cell Infect Microbiol. 2023;13:1132495.
35. Spengler JR, Bergeron E, Rollin PE. Seroepidemiological Studies of Crimean-Congo Hemorrhagic Fever Virus in Domestic and Wild Animals. PLoS Negl Trop Dis. 2016;10(1):e0004210.
36. Mostafavi E, Haghdoost A, Khakifirouz S, Chinikar S. Spatial analysis of Crimean Congo hemorrhagic fever in Iran. Am J Trop Med Hyg. 2013;89(6):1135-41.
37. Humolli I, Dedushaj I, Zupanac TA, Mucaj S. Epidemiological, serological and herd immunity of Crimean-Congo haemorrhagic fever in Kosovo. Med Arh. 2010;64(2):91-3.
38. Papa A, Sidira P, Kallia S, Ntouska M, Zotos N, Doumbali E, et al. Factors associated with IgG positivity to Crimean-Congo hemorrhagic fever virus in the area with the highest seroprevalence in Greece. Ticks Tick Borne Dis. 2013;4(5):417-20.

39. Yen YC, Kong LX, Lee L, Zhang YQ, Li F, Cai BJ, et al. Characteristics of Crimean-Congo hemorrhagic fever virus (Xinjiang strain) in China. Am J Trop Med Hyg. 1985;34(6):1179-82.
40. Fisher-Hoch SP, McCormick JB, Swanepoel R, Van Middlekoop A, Harvey S, Kustner HG. Risk of human infections with Crimean-Congo hemorrhagic fever virus in a South African rural community. Am J Trop Med Hyg. 1992;47(3):337-45.
41. Zeller HG, Cornet JP, Camicas JL. Experimental transmission of Crimean-Congo hemorrhagic fever virus by west African wild ground-feeding birds to Hyalomma marginatum rufipes ticks. Am J Trop Med Hyg. 1994;50(6):676-81.
42. Rodriguez LL, Maupin GO, Ksiazek TG, Rollin PE, Khan AS, Schwarz TF, et al. Molecular investigation of a multisource outbreak of Crimean-Congo hemorrhagic fever in the United Arab Emirates. Am J Trop Med Hyg. 1997;57(5):512-8.
43. Sorvillo TE, Rodriguez SE, Hudson P, Carey M, Rodriguez LL, Spiropoulou CF, et al. Towards a Sustainable One Health Approach to Crimean-Congo Hemorrhagic Fever Prevention: Focus Areas and Gaps in Knowledge. Trop Med Infect Dis. 2020;5(3).
44. zawya.com. Oman shuts some livestock farms amid disease fears. Available from: https://www.zawya.com/en/life/oman-shuts-some-livestock-farms-amid-disease-fears-h1ytbf1d [Cited 2023-08-03]. ZAWYA by Refinitiv Middle East. 2016.
45. Sadaqat M. Ban imposed on slaughtering of animals in Haripur. Available from: https://tribune.com.pk/story/1106615/congo-virus-ban-imposed-slaughtering-animals-haripur [Cited 2023-08-03]. The Express Tribune. 2016.
46. alsumaria.tv. [An Iraqi governorate prevents the entry of livestock due to an outbreak of hemorrhagic fever (in Arabic)]. Available from: https://tinyurl.com/d7bmmm2k [Cited 2023-08-03]. Al-Sumaria News. 2023.
47. alsabaah.iq. [Agriculture warns of exacerbation of hemorrhagic fever (in Arabic)]. Available from: https://alsabaah.iq/79989-.html [Cited 2023-08-03]. Al Sabah Newspaper. 2023.
48. Tannenbaum J, Bennett BT. Russell and Burch's 3Rs then and now: the need for clarity in definition and purpose. J Am Assoc Lab Anim Sci. 2015;54(2):120-32.
49. Hubrecht RC, Carter E. The 3Rs and Humane Experimental Technique: Implementing Change. Animals (Basel). 2019;9(10).
50. Pickering BS, Smith G, Pinette MM, Embury-Hyatt C, Moffat E, Marszal P, et al. Susceptibility of Domestic Swine to Experimental Infection with Severe Acute Respiratory Syndrome Coronavirus 2. Emerg Infect Dis. 2021;27(1):104-12.
51. Cross RW, Prasad AN, Borisevich V, Geisbert JB, Agans KN, Deer DJ, et al. Crimean-Congo hemorrhagic fever virus strains Hoti and Afghanistan cause viremia and mild clinical disease in cynomolgus monkeys. PLoS Negl Trop Dis. 2020;14(8):e0008637.
52. Kozak RA, Fraser RS, Biondi MJ, Majer A, Medina SJ, Griffin BD, et al. Dual RNA-Seq characterization of host and pathogen gene expression in liver cells infected with Crimean-Congo Hemorrhagic Fever Virus. PLoS Negl Trop Dis. 2020;14(4):e0008105.
53. Bente DA, Alimonti JB, Shieh WJ, Camus G, Stroher U, Zaki S, et al. Pathogenesis and immune response of Crimean-Congo hemorrhagic fever virus in a STAT-1 knockout mouse model. J Virol. 2010;84(21):11089-100.
54. Li H, Smith G, Goolia M, Marszal P, Pickering BS. Comparative characterization of Crimean-Congo hemorrhagic fever virus cell culture systems with application to propagation and titration methods. Virol J. 2023;20(1):128.
55. woah.org. Classical swine fever (infection with classical swine fever virus). Available from: https://www.woah.org/fileadmin/Home/eng/Health_standards/tahm/3.09.03_CSF.pdf [Cited 2023-08-10]. World Organisation for Animal Health. 2022.

56. Deyde VM, Khristova ML, Rollin PE, Ksiazek TG, Nichol ST. Crimean-Congo hemorrhagic fever virus genomics and global diversity. J Virol. 2006;80(17):8834-42.
57. Wick R. rrwick/Porechop. Available from: https://github.com/rrwick/Porechop [Cited 2023-12-23]. GitHub, Inc. 2020.
58. De Coster W, Rademakers R. NanoPack2: population-scale evaluation of long-read sequencing data. Bioinformatics. 2023;39(5).
59. Li H. Minimap2: pairwise alignment for nucleotide sequences. Bioinformatics. 2018;34(18):3094-100.
60. Katoh K, Standley DM. MAFFT multiple sequence alignment software version 7: improvements in performance and usability. Mol Biol Evol. 2013;30(4):772-80.
61. Schwarz TF, Nsanze H, Longson M, Nitschko H, Gilch S, Shurie H, et al. Polymerase chain reaction for diagnosis and identification of distinct variants of Crimean-Congo hemorrhagic fever virus in the United Arab Emirates. Am J Trop Med Hyg. 1996;55(2):190-6.
62. Tonbak S, Aktas M, Altay K, Azkur AK, Kalkan A, Bolat Y, et al. Crimean-Congo hemorrhagic fever virus: genetic analysis and tick survey in Turkey. J Clin Microbiol. 2006;44(11):4120-4.
63. Rodriguez SE, Hawman DW, Sorvillo TE, O'Neal TJ, Bird BH, Rodriguez LL, et al. Immunobiology of Crimean-Congo hemorrhagic fever. Antiviral Res. 2022;199:105244.
64. Zivcec M, Safronetz D, Scott D, Robertson S, Ebihara H, Feldmann H. Lethal Crimean-Congo hemorrhagic fever virus infection in interferon alpha/beta receptor knockout mice is associated with high viral loads, proinflammatory responses, and coagulopathy. J Infect Dis. 2013;207(12):1909-21.
65. Lehman HK, Segal BH. The role of neutrophils in host defense and disease. J Allergy Clin Immunol. 2020;145(6):1535-44.
66. Russo RC, Garcia CC, Teixeira MM, Amaral FA. The CXCL8/IL-8 chemokine family and its receptors in inflammatory diseases. Expert Rev Clin Immunol. 2014;10(5):593-619.
67. Cassatella MA. Neutrophil-derived proteins: selling cytokines by the pound. Adv Immunol. 1999;73:369-509.
68. Kaiser R, Leunig A, Pekayvaz K, Popp O, Joppich M, Polewka V, et al. Self-sustaining IL-8 loops drive a prothrombotic neutrophil phenotype in severe COVID-19. JCI Insight. 2021;6(18).
69. Brandes M, Klauschen F, Kuchen S, Germain RN. A systems analysis identifies a feedforward inflammatory circuit leading to lethal influenza infection. Cell. 2013;154(1):197-212.
70. Metzemaekers M, Gouwy M, Proost P. Neutrophil chemoattractant receptors in health and disease: double-edged swords. Cell Mol Immunol. 2020;17(5):433-50.
71. Lum G, Leal-Khouri S. Significance of low serum urea nitrogen concentrations. Clin Chem. 1989;35(4):639-40.
72. Hosten AO. BUN and Creatinine.. In: Clinical Methods: The History, Physical, and Laboratory Examinations. 3rd edition. Boston: Butterworths. 1990.
73. Constable PD, Hinchcliff KW, Done SH, Grünberg W. Diseases of the Liver. In: Veterinary Medicine. 11th Edition. Elsevier Ltd. 2017.
74. Lim LM, Kuo HT, Kuo MC, Chiu YW, Lee JJ, Hwang SJ, et al. Low serum calcium is associated with poor renal outcomes in chronic kidney disease stages 3-4 patients. BMC Nephrol. 2014;15:183.
75. Mizushiri S, Daimon M, Murakami H, Kamba A, Osonoi S, Yamaichi M, et al. Lower serum calcium levels are a risk factor for a decrease in eGFR in a general non-chronic kidney disease population. Sci Rep. 2018;8(1):14213.
76. Yu H, Zhou D, Jia W, Guo Z. Locating the source of hyperglycemia: liver versus muscle. J Diabetes. 2012;4(1):30-6.
77. Mehta RL. Glycemic control and critical illness: is the kidney involved? J Am Soc Nephrol. 2007;18(10):2623-7.

78. Gonzalez JP, Camicas JL, Cornet JP, Wilson ML. Biological and clinical responses of west African sheep to Crimean-Congo haemorrhagic fever virus experimental infection. Res Virol. 1998;149(6):445-55.
79. Fels JM, Maurer DP, Herbert AS, Wirchnianski AS, Vergnolle O, Cross RW, et al. Protective neutralizing antibodies from human survivors of Crimean-Congo hemorrhagic fever. Cell. 2021;184(13):3486-501 e21.
80. Griffin DE. Why does viral RNA sometimes persist after recovery from acute infections? PLoS Biol. 2022;20(6):e3001687.
81. Ramakrishnan RK, Kashour T, Hamid Q, Halwani R, Tleyjeh IM. Unraveling the Mystery Surrounding Post-Acute Sequelae of COVID-19. Front Immunol. 2021;12:686029.
82. Miller KD, Matullo CM, Milora KA, Williams RM, O'Regan KJ, Rall GF. Immune-Mediated Control of a Dormant Neurotropic RNA Virus Infection. J Virol. 2019;93(18).
83. Fragkoudis R, Dixon-Ballany CM, Zagrajek AK, Kedzierski L, Fazakerley JK. Following Acute Encephalitis, Semliki Forest Virus is Undetectable in the Brain by Infectivity Assays but Functional Virus RNA Capable of Generating Infectious Virus Persists for Life. Viruses. 2018;10(5).
84. Levine B, Griffin DE. Persistence of viral RNA in mouse brains after recovery from acute alphavirus encephalitis. J Virol. 1992;66(11):6429-35.
85. Appler KK, Brown AN, Stewart BS, Behr MJ, Demarest VL, Wong SJ, et al. Persistence of West Nile virus in the central nervous system and periphery of mice. PLoS One. 2010;5(5):e10649.
86. Mathur A, Kulshreshtha R, Chaturvedi UC. Induction of secondary immune response by reactivated Japanese encephalitis virus in latently infected mice. Immunology. 1987;60(4):481-4.
87. Harrower J, Kiedrzynski T, Baker S, Upton A, Rahnama F, Sherwood J, et al. Sexual Transmission of Zika Virus and Persistence in Semen, New Zealand, 2016. Emerg Infect Dis. 2016;22(10):1855-7.
88. Turmel JM, Abgueguen P, Hubert B, Vandamme YM, Maquart M, Le Guillou-Guillemette H, et al. Late sexual transmission of Zika virus related to persistence in the semen. Lancet. 2016;387(10037):2501.
89. Keita AK, Koundouno FR, Faye M, Dux A, Hinzmann J, Diallo H, et al. Resurgence of Ebola virus in 2021 in Guinea suggests a new paradigm for outbreaks. Nature. 2021;597(7877):539-43.
90. Nelson AN, Lin WW, Shivakoti R, Putnam NE, Mangus L, Adams RJ, et al. Association of persistent wild-type measles virus RNA with long-term humoral immunity in rhesus macaques. JCI Insight. 2020;5(3).
91. Lin WW, Moran E, Adams RJ, Sievers RE, Hauer D, Godin S, et al. A durable protective immune response to wild-type measles virus infection of macaques is due to viral replication and spread in lymphoid tissues. Sci Transl Med. 2020;12(537).
92. Horta-Barbosa L, Hamilton R, Wittig B, Fuccillo DA, Sever JL, Vernon ML. Subacute sclerosing panencephalitis: isolation of suppressed measles virus from lymph node biopsies. Science. 1971;173(3999):840-1.
93. ter Meulen V, Muller D, Kackell Y, Katz M, Meyermann R. Isolation of infectious measles virus in measles encephalitis. Lancet. 1972;2(7788):1172-5.
94. Haspel MV, Knight PR, Duff RG, Rapp F. Activation of a latent measles virus infection in hamster cells. J Virol. 1973;12(4):690-5.
95. Li H, Omange RW, Liang B, Toledo N, Hai Y, Liu LR, et al. Vaccine targeting SIVmac251 protease cleavage sites protects macaques against vaginal infection. J Clin Invest. 2020;130(12):6429-42.
96. Li H, Omange RW, Plummer FA, Luo M. A novel HIV vaccine targeting the protease cleavage sites. AIDS Res Ther. 2017;14(1):51.
97. Luo M. Natural Immunity against HIV-1: Progression of Understanding after Association Studies. Viruses. 2022;14(6).
